# The AIMAR recommendations for early diagnosis of chronic obstructive respiratory disease based on the WHO/GARD model*

**DOI:** 10.1186/2049-6958-9-46

**Published:** 2014-09-03

**Authors:** Stefano Nardini, Isabella Annesi-Maesano, Mario Del Donno, Maurizio Delucchi, Germano Bettoncelli, Vincenzo Lamberti, Carlo Patera, Mario Polverino, Antonio Russo, Carlo Santoriello, Patrizio Soverina

**Affiliations:** 1 Pulmonary and TB Unit, Vittorio Veneto General Hospital, Vittorio Veneto, TV, Italy; 2 EPAR, INSERM UMRS-1136 IPLESP, Paris, France; 3 EPAR, Paris Université Pierre et Marie Curie, UMRS-1136 IPLESP, Paris, France; 4 Respiratory Unit, “G. Rummo” Hospital, Benevento, Italy; 5 Internal Medicine Unit , Saluzzo Hospital, ASL CN1 Regione Piemonte, Saluzzo, CN, Italy; 6 General Practitioner, Brescia, SIMG Area Respiratoria, Florence, Italy; 7 Sport Medicine, ULSS 7 Regione Veneto, Vittorio Veneto TV, Italy; 8 General Practitioner, Regione Veneto, San Donà di Piave, VE, Italy; 9 Provincial Respiratory Pole, ASL Salerno, Salerno, Italy; 10 Respiratory Unit, “G. Rummo” Hospital, Benevento, Italy; 11 Respiratory Function Unit, Polla Hospital, ASL Salerno Salerno, Italy; 12 General Practitioner, Naples, SIMG Area Respiratoria, Naples, Italy

**Keywords:** COPD, Early diagnosis, Guidelines, Prevention, Respiratory diseases

## Abstract

Respiratory diseases in Italy already now represent an emergency (they are the 3^rd^ ranking cause of death in the world, and the 2^nd^ if Lung cancer is included). In countries similar to our own, they result as the principal cause for a visit to the general practitioner (GP) and the second main cause after injury for recourse to Emergency Care. Their frequency is probably higher than estimated (given that respiratory diseases are currently underdiagnosed). The trend is towards a further increase due to epidemiologic and demographic factors (foremost amongst which are the widespread diffusion of cigarette smoking, the increasing mean age of the general population, immigration, and pollution). Within the more general problem of chronic disease care, chronic respiratory diseases (CRDs) constitute one of the four national priorities in that they represent an important burden for society in terms of mortality, invalidity, and direct healthcare costs. The strategy suggested by the World Health Organization (WHO) is an integrated approach consisting of three goals: inform about health, reduce risk exposure, improve patient care. The three goals are translated into practice in the three areas of prevention (1-primary, 2-secondary, 3-tertiary) as: 1) actions of primary (universal) prevention targeted at the general population with the aim to control the causes of disease, and actions of Predictive Medicine - again addressing the general population but aimed at measuring the individual’s risk for disease insurgence; 2) actions of early diagnosis targeted at groups or - more precisely - subgroups identified as at risk; 3) continuous improvement and integration of care and rehabilitation support - destined at the greatest possible number of patients, at all stages of disease severity. In Italy, COPD care is generally still inadequate. Existing guidelines, institutional and non-institutional, are inadequately implemented: the international guidelines are not always adaptable to the Italian context; the document of the Agency for Regional Healthcare Services (AGE.NA.S) is a more suited compendium for consultation, and the recent joint statement on integrated COPD management of the three major Italian scientific Associations in the respiratory area together with the contribution of a Society of General Medicine deals prevalently with some critical issues (appropriateness of diagnosis, pharmacological treatment, rehabilitation, continuing care); also the document “Care Continuity: Chronic Obstructive Pulmonary Disease (COPD)” of the Global Alliance against chronic Respiratory Diseases (GARD)-Italy does not treat in depth the issue of early diagnosis. The present document – produced by the AIMAR (Interdisciplinary Association for Research in Lung Disease) Task Force for early diagnosis of chronic respiratory disease based on the WHO/GARD model and on available evidence and expertise –after a general examination of the main epidemiologic aspects, proposes to integrate the above-mentioned existing documents. In particular: a) it formally indicates on the basis of the available evidence the modalities and the instruments necessary for carrying out secondary prevention at the primary care level (a pro-active,‘case-finding’approach; assessment of the individual’s level of risk of COPD; use of short questionnaires for an initial screening based on symptoms; use of simple spirometry for the second level of screening); b) it identifies possible ways of including these activities within primary care practice; c) it places early diagnosis within the “systemic”, consequential management of chronic respiratory diseases, which will be briefly described with the aid of schemes taken from the Italian and international reference documents.

## Review

### Defining the responsibilities

The United Nations in 2011 identified chronic non-communicable diseases as the health priority of this decade [1]. Also the World Health Organization (WHO) defines chronic diseases as a global threat and includes among the major diseases - besides cancer, cardiovascular diseases and diabetes - also chronic respiratory diseases, chiefly COPD [2]. According to the WHO documents [3] the epidemic of chronic diseases can be combatted only through prevention (primary and secondary) and an integrated treatment of the disease after the diagnosis.

The means proposed include firstly the development of national programs of prevention and control of chronic respiratory diseases, aimed not only at defining the most pertinent healthcare strategies and actions, but also at raising political and social interest about this issue of public health. Alongside these initiatives also fundamental are the awareness and health education of the general public (for primary and secondary prevention) and training of healthcare professionals (not only in responding to citizens’ needs regarding primary and secondary prevention, but also for appropriate treatment to the patients, a treatment that must integrate the various points of view of the stakeholders).

Adherence to treatment and integrated care are considered the best means to reduce inappropriate treatment practice, which represents not only a cost for society but also a risk for the individual. One of the most striking examples of such costs are admissions to Emergency Care, which, in the United Kingdom, show COPD as ranking 1^st^ among the chronic diseases and asthma 3^rd^ as a cause for recourse to Emergency Care [4].

To attain these goals - fundamental for an optimal care of all chronic diseases - the organizational requirements are as follows: a) a greater role played by general practitioners (GPs); b) creation of intermediate levels of care between primary care and the hospital (involving better and faster communication between the specialist and GP - these last organized possibly in some form of association - as well as a more rational use of telemedicine); and c) structuring of home and community care, which includes also redefining the role of the hospital for acute patients and the creation of intermediate inpatient facilities between the hospital and the patient’s home [5].

Specifically concerning COPD - and in general valid for almost all chronic respiratory diseases - the actions to be implemented have been identified in a document of the United Kingdom published a few years ago that established the priorities for the National Health Service as follows: 1. prevent the development of COPD through a significant reduction of the number of smokers in the community; 2. improve diagnosis of COPD, in particular through a more widespread use of spirometry tests; 3. help patients to self-manage their own disease through respiratory rehabilitation; 4. integrate the care of patients affected by COPD, i.e. link specialist care to primary care [6].

In accordance with the above, in COPD - as for chronic respiratory diseases generally - the State and National Health Service are responsible for planning (and funding) the most appropriate actions for the various levels of preventive action (some of which are common to all other chronic diseases) and for integrating the healthcare services. It is the responsibility of health professionals (and their Scientific Societies) to know about and apply the fundamentals of prevention and treatment including their relative organizational aspects. General Population have the responsibility to learn about the behaviors that promote health ("health education"), as well as the characteristics of the early phases of respiratory diseases (so they can confirm or disconfirm their existence through the appropriate actions of secondary prevention). Patients Associations should help associates (both patients and relatives or caregivers) in the educational process for self-management of disease once it is manifest ("education about the disease") as well as advocate for public and political awareness of the burden of the disease.

## The size of the problem and consequent strategies

In Italy, as there is no register for such diseases nor a corresponding exemption ticket, precise and reliable official data concerning COPD are lacking. Moreover, and not only in Italy, a large underestimation of the disease itself has been demonstrated.

In Italy in 2001 - based on WHO data - out of a total 556,892 deaths 64,984 were due to respiratory causes: of these, 31,968 deaths were due to lung cancer, 16,295 to chronic obstructive respiratory diseases, 8,377 to pneumonia and 8,344 to other respiratory causes. In 2006, there were in Italy (excluding deaths due to lung cancer) 35,751 deaths due to respiratory causes (57% males) representing 6.4% of all deaths. In 2008, 37,659 deaths were registered (6.5% of the total mortality). Of these, the deaths due to asthma were 474, while those due to COPD, chronic bronchitis, emphysema and other chronic obstructive diseases were 20,786 [7]. Respiratory diseases all together thus represent the 3^rd^ ranking cause of mortality (or 2^nd^, if lung cancer is included).

These are deaths that mostly affect the more advanced age-groups, and approximately half of them are due to COPD; nevertheless, when the moment of death arrives the patient will have already lived many years of invalidity as shown by the European Community Respiratory Health Survey (ECRHS) which highlighted that in the 20-44 years age-group new cases of COPD per 1,000 inhabitants each year range from a little more than 1 to almost 3 depending on the criterion of obstruction utilized [8].

On the basis of the National Institute of Statistics (ISTAT) data, about 5% of adult Italian males are allegedly affected by COPD and about 4% of women, but as noted clearly in the COPD guidelines of AGE.NA.S: “This estimate, that places COPD in 6^th^ position of chronic diseases present in Italy and which, translated into an absolute value, identifies more than 2,600,000 Italian citizens as affected is almost certainly an underestimate of the real dimensions of the prevalence of the disease” [9].

This statement is confirmed by a recent Italian study that compared the data of prevalence drawn from administrative records with the general medicine database (Health Search) and with estimates based on ISTAT data. This study, while it showed good agreement between the three sources concerning diabetes, heart failure and ischemic heart disease, revealed COPD prevalence based on administrative data to be markedly lower with respect to the data from general medicine [10].

More in line with the picture that emerges from the international literature are the data of Viegi et al., on the basis of which 9.9% of people between 25 and 45 years suffer from obstructive disorders while in the overall age-range of 25-73 years 11%, 18% and 40% of people are affected depending on whether one applies, respectively, the criteria of the European Respiratory Society (ERS), clinical criteria, or those of the American Thoracic Society (ATS) [11].

Slightly lower prevalences have been reported in the past by the Italian GPs (SIMG) network (Health Search), which shows 4.5% of the general population as affected by COPD, and well over 8% of males and 4% of females aged over 65 years [12].

Concerning the different levels of disease severity and according to the previous GOLD staging, Viegi’s group showed the COPD prevalence in Italy as follows: for males, 14.2% = stage 0 or at risk (people with chronic cough or sputum production), 12.3% = stage I or mild, 4.5% = stage II or moderate, 0.4% = stage III-IV or severe-very severe; for females, 10.1% = stage 0 or at risk (with chronic cough or sputum production), 7.3% = stage I or mild, 2.2% = stage II or moderate, 0.3% = stage III-IV or severe-very severe. Similar results have been found also in other countries [13].

These are figures that evidence an epidemic trend, which needs to be faced as a real emergency. An emergency all the greater considering that - as said before - this frequency is high notwithstanding the figures are underestimated [14]. Underestimation derives mostly from the fact that a disease with a low profile or visibility is also less often suspected (compared to other more “publicized” diseases) and consequently less often investigated diagnostically. A classic example are patients who report dyspnea on exercise: they are almost always referred for an electrocardiogram or to a cardiac specialist and consultation by a pulmonologist comes only at a second or even third step. Patients with COPD (but also asthma) are often undertreated to the point that not even those affected know they have a chronic obstructive disease [15]. When one compares patients’ awareness about their disease with that of other chronic pathologies, such as diabetes or hypertension, one sees that only a fifth (20.6%) of patients with COPD are aware about their disease compared to 94.3% of diabetic patients, 69% of patients with hypertension and 57.2% of heart disease patients [16]. Of note, in the same study, only slightly more than 17% of patients affected by COPD remembered having received advice to quit smoking.

Underdiagnosis has mostly been investigated by studies abroad that report percentages of missed COPD diagnosis ranging between 75% and 91% in Sweden [13] and 80% in the United Kingdom [17,18]. Strange as it may seem, even severe cases, despite the fact they provoke significant respiratory symptoms, are diagnosed in only half of the patients. The studies just mentioned found in fact that missed diagnosis of COPD regarded 50% of severe cases, 81% of moderate cases and more than 99% of mild cases in Sweden, while in the United Kingdom only 46.8% of cases of severe or very severe COPD had received a diagnosis of respiratory disease.

In Italy, a recent qualitative study that used the approach recommended by GARD/WHO, i.e. social communication - with posters and leaflets placed in the GP’s office and television ads - and offering a free spirometry to all who considered they had symptoms of respiratory disease, found chronic lung disease to be present in 356 (86%) out of 414 people who presented spontaneously, of whom 320 (77% of the total and 90% of those affected) were without diagnosis. Interestingly, only 6% (13 of the 221 people found with COPD) were already known to have COPD [19].

Besides the above-mentioned studies, others have reported, in large-size samples with “functional” confirmation of the clinical suspicion, a prevalence of bronchial obstruction in adults in industrialized countries of up to 16% [20], but which a recent review has shown can reach as much as 37% depending on the group examined and the criterion used for diagnosis of COPD and of bronchial obstruction [21]. The previously cited study [18] carried out on more than 8,000 British adults over 35 years of age showed a prevalence of COPD (defined with spirometry) of 13.3%. Overall, the most reliable estimates for the global burden of COPD range from 3.6% to 10.1% [21], from 4% to 10% [22], and from 9% to 10% [23]. While acknowledging that COPD prevalence can vary in terms of the mean age of the population, the prevalence of smokers and the spirometry criteria used, it can be concluded that the prevalence of COPD in developed countries - and hence also in ours - can be estimated at probably 10% or more in individuals over 45 years of age, with a cumulative incidence of 5% of new cases (according to GOLD criteria) over a period of seven years in individuals aged 45-79 years, previously healthy from the respiratory point of view [24].

If the first cause for missed diagnosis of this respiratory disease is low patient sensitivity about the symptoms themselves of the disease, when symptoms finally are reported to the doctor scant use is made by the latter of the procedures that could correctly lead to diagnosis. In a study carried out a few years ago, only about half of patients who reported symptoms performed a spirometry test [22]. The situation is worse still in Italy: based on the aforementioned Health Search data, only about 30% of the cases of (recorded) COPD diagnosis are accompanied by a spirometry test [25]. Also in the hospital setting the percentage of COPD diagnosis with spirometry documentation is decidedly lower than expectations, with mean values around 15% in internal medicine departments [26] and around 60% in the pulmonary setting [27].

Besides being harmful for the individual affected, this underestimate and under-awareness involves a cost for society (for late diagnosis and inappropriate treatment which in turn lead to a not-inevitable number of cases of invalidity) and a drain on healthcare resources (for inadequate or nonexistent planning of specific patient care - in Italy there is still no exemption for medical costs related to COPD). In fact the costs of non-programmed care - a consequence of underdiagnosis - can double those of programmed care [28].

Last, it is worthwhile noting that COPD care already now represents a high cost for the community: it is the 7^th^ ranking cause for number of admissions and for cost of hospital stay, and the 2^nd^ for mean length of stay, with a percentage of hospital admissions that represents 1.2% of the total. Hospitalizations for COPD constitute more than 6.5% of the total; based on U.K. data [29] respiratory diseases constitute the leading cause for visits to the GP, coming before even those for musculoskeletal problems. Also based on the same source, respiratory diseases represent the 2^nd^ cause for access to the Emergency Ward, after accidental lesions (wounds, injuries and poisoning) [29]. A patient treated at home with long-term O_2_ therapy costs approximately 4,500 euro per year [30].

The general prevalence of chronic respiratory diseases and hence the cost of their care are destined to rise, given that such diseases and the relative consequences are events associated with the elderly and, moreover, that advances in healthcare are improving patients’ mean survival.

In conclusion, using with prudence the above-mentioned estimates (i.e. from 4 to 10% of the population) about 3 millions people would be affected by this disease, i.e. more or less the same number as those affected by diabetes (4.9% of the general population: 5.0% of females, 4.7% of males), i.e. almost 3 million people [31] - a chronic disease that benefits from very different medical and social funding and a very different attention on the part of health professionals. It is not by chance that WHO recommends the adoption of initiatives to raise awareness about the epidemiological importance of COPD through advocacy both of the wider public and of politicians and decision-makers in general [3].

## Global management of COPD

As said, the management of COPD and chronic respiratory diseases generally needs to be put in the context of today’s healthcare in which chronic invalidating diseases have an overwhelming prevalence (cancer, diabetes and cardiac disease as well as respiratory diseases). In addition to the increase in the number of people affected by these diseases due, as already mentioned, to the growing average age of the population, the cost of their care is rising due to the development of new, effective pharmacological treatments and new rehabilitation protocols that increase still further the life expectancy. These trends place the accent on the sustainability of the healthcare budget: if today there is 1 person over 65 years for every 2 people of an employable age, this ratio is destined to become inverted in the short term and we will have 2 elderly for every 1 person of an employable age. For this reason, in the U.K. - which has a national health system (NHS) similar to our own - a debate has arisen about reorganizing the health system in relation to chronic respiratory disease [32]: the NHS should in future help citizens to choose a healthier lifestyle through health education and information, promoting a healthy “air” environment, and offering them integrated services of primary, secondary and tertiary prevention.

In practice, a national health service should, while ensuring care for already existing chronic diseases (with the aim to reduce their invalidating consequences through early diagnosis and rehabilitation), implement actions aimed at preventing the onset of new ones. Part of the care for currently existing diseases is an optimization of the existing services (which includes a reduction of the inappropriate healthcare practices) and their integration. By way of example, currently care for chronic diseases is prevalently hospital-based, but it will have to organize itself differently to become prevalently based in the local community. Also in Italy we have begun to face this problem with government document “Gaining health” (“Guadagnare salute”) produced in the spring of 2007 that highlights the importance of prevention in community health management (at all levels) and the relative strategies and consequent working actions to achieve it [33].

In Italy, as in the U.K., the possible remedies at organizational level are:a greater involvement of GPs and the creation of intermediate levels between the assessment carried out in the GP’s office and hospital, with more frequent and faster communication between specialist and GP (ideally the GP should be located in an office in association with other GPs); a rationally designed use of telemedicine; and a better structuring of homecare, with a greater presence and integration of community nurses. GARD-Italy, under the auspices of our Ministry, highlights also the importance of fighting risk factors, of anticipating the diagnosis and, thus, of a system of care aimed at preventing/delaying complications, relapses and progression of the disease [34].

**Figure 1:**
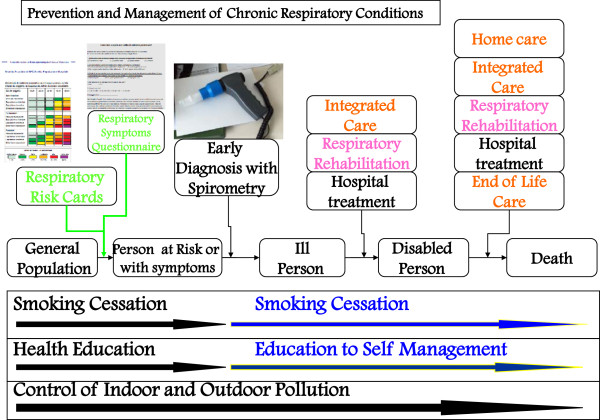
**General flow chart for COPD management.** From [35] mod.

A way to put into practice the general principles outlined above is exemplified in the flow diagram in Figure 1 modified from the joint statement of the three leading scientific societies in Respiratory Medicine in conjunction with a scientific society of General Medicine, *The Integrated Management of COPD*[35]. The cited document and relative flow diagram were used - together with the Italian AGE.NA.S guidelines on COPD [36] and with the documents of GARD/WHO and GARD-Italy (see below) as a basis for elaborating the model in the present document.

The flow chart represents the general management of COPD and can serve for the management of any chronic respiratory condition (accordingly modifying the instruments for early diagnosis). The general population must be stimulated and assisted to keep healthy: this is achieved through health education (which means guiding them to choose healthier behaviors) together with the elimination of outdoor air pollution (through appropriate legislation and continuous monitoring of air quality), education about indoor pollution control, and tobacco control.

It is possible to identify in the general population individuals who, as bearers of fragility or defects or particular characteristics, have a predisposition to develop disease: identifying them allows the maximum individual tailoring of interventions (so-called “predictive” medicine). For example, as interventions of predictive medicine one can intend actions addressed at evaluating cholesterolemia in the prevention of cardiovascular diseases. Concerning COPD, the best available tool for identifying persons most at risk are the respiratory risk cards produced by the National Research Center (CNR) and National Institute of Health [37].

Persons identified through the use of risk cards - in one of the possible modes (see below) - or those who due to presence of respiratory symptoms - even generic - are suspected of COPD undergo an early diagnosis procedure based on spirometry as the method of choice. Once the diagnosis of COPD is made the patient enters a treatment process based on self-management, aided by education about the therapy, programmed integration between hospital and local community, and pulmonary rehabilitation (PR), the goal of which is to delay the invalidating consequences of the disease. With the progressive worsening of the disease, home care must be organized and, in the final phases, pain care and end-of-life care. Through these organizational modalities, and only in this way, COPD care (and that of the other chronic diseases) is based on an appropriate use of resources and is sustainable by the community, as demonstrated by the U.K. hospitals clinical audit system [38].

All points presented in the flow chart - in particular the issue of early diagnosis - will be discussed in the following paragraphs.

### Primary prevention of COPD

**Figure 2:**
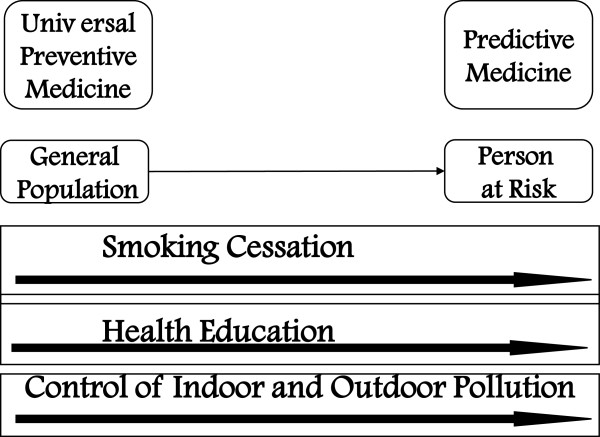
Interventions for primary prevention of COPD.

Primary prevention signifies prevention of the occurrence of chronic respiratory disease. This is achieved principally, but not only, through a consistent reduction of the number of smokers in the community, together with health education and indoor and outdoor pollution control (Figure 2).

In fact the WHO estimates that only about 42% of the global epidemiologic burden of COPD is due to environmental exposure: in particular, occupational exposure would account for 12%, and domestic exposure to home cooking fuels for 22% (bear in mind that these are estimates regarding the whole world for which there are obviously different living conditions from those characteristic of the western world, in which this exposure is far less important). Outdoor pollution is estimated to be responsible for 3% of cardiopulmonary mortality. On the contrary, the preponderant role of smoking is underlined by the same WHO estimates: for COPD active smoking alone contributes to about 38% of the global burden [39].

Reduction in the number of smokers is brought about, at the level of the general population, by legislative and commercial measures (bans, sales limitations, taxation) rather than through health treatments. These latter, in particular the most individually tailored ones (and, where necessary, the most “intensive”) are reserved for those within the general population who on account of their characteristics (genetic, habit- or lifestyle-related) are at greater risk than others. Particular effort hence will be spent on identifying them (predictive medicine) and directing them *a priori* to the appropriate care.

In reducing the number of smokers one acts not only on the principal cause of COPD but also on the principal risk factor of other prevalent chronic diseases. Smoking in fact contributes overall to about 12% of the risk expressed in disability adjusted life years (DALYs), hypertension being responsible for approximately 9%, overweight for 8%, alcohol and cholesterol for about 6%, and physical inactivity for 3%.

In the general population tobacco control is to be understood as a primary prevention measure, i.e. aimed at avoiding the onset of diseases linked to smoking itself, and it should be held quite distinct from the role that smoking control has in the people already affected by disease (see section How to organize treatment: smoking cessation and related pharmaceutical drugs). The control of this cause of diseases is the most important measure in the field of prevention of chronic diseases and can be achieved either by impeding smoking initiation of adolescents not yet smokers or by making already active smokers quit smoking *before* they develop a disease. The two approaches are not alternatives but, rather, complement each other; nonetheless of the two strategies the one that obtains best results in the short term is smoking cessation of active smokers. In fact, while the health benefits of non-initiation will be seen after approximately one generation (i.e. after about 20 years), within just a few days of smoking cessation the ex-smoker enjoys health benefits, in particular cardiovascular benefits, the full effects of which are revealed over a period of about 16 years. Essential for the success of both approaches is that they are implemented in a social and cultural context that is able to control smoking from all points of view - legislative, commercial and price regulation - focused on protecting the health of non-smokers. The guide for all these actions is the Framework Convention on Tobacco Control (FCTC) of the WHO, a convention to which also Italy has adhered (for further details, please refer to [40]). The main points of the strategy are summed up by the acronym MPOWER (Table 1) which refers to the document [41]. The offer of services for smoking cessation (made obligatory by article 14 of the FCTC) permits - as shown above - to act for the prevention not only of chronic respiratory diseases but also of chronic diseases other than respiratory which represent the great majority of the causes of death and invalidity in our community.

**Table 1:** Strategy for smoking control according to the Framework Convention on Tobacco Control (FCTC) of the WHO

**Objective 3: To promote interventions to reduce the main modifiable risk factors e.g. tobacco use**	
**Key areas of actions for tobacco use reduction**	
●	**M**onitor tobacco use through tobacco prevention policies
●	**P**rotect people from tobacco smoke in public places and workplaces
●	**O**ffer help to people who want to stop
●	**W**arn people about dangers of tobacco
●	**E**nforce bans of advertising, promotion and sponsorship
●	**R**aise tobacco taxes and prices

From [41], mod.

Both prevention of smoking initiation in young non-smokers and smoking cessation in adult smokers represent effective strategies of prevention of COPD (and of other smoking-related diseases). However, as shown above, prevention of initiation yields results after 20-40 years, which is the mean time interval between the beginning of exposure to harm from smoking and the onset of a disease correlated to it.

As said at the start, it is also important - though less - to control outdoor and indoor air pollution. This theme is intertwined with the more general one of health promotion [42] which is not simply healthcare but the result of coordination between all sectors and subjects whose activity is fundamental for the health of the community. In these fields of health promotion the role of the health services is definitely marginal accounting for about 15% compared with socio-economic factors and lifestyle (which account for about 50%), the environment (about 20%) and genetic heredity (15%). For this reason one speaks of “intersectorial” interventions of health promotion to underline the role, for example, of control of pollution from vehicles brought about through advances in the design of internal combustion engines or through the modification of urban and transportation policies.

For what concerns indoor pollution, a distinction should be made between that of the workplace and that of the living environment. In the first case legislation and control based on sanctions play a leading role, in that they can regulate the exposure of workers to substances that are potentially dangerous for the respiratory system. Concerning the home and living environment, while in public spaces legislation and repression still have a role, for the home environment only health education is valid. One should bear in mind that the chief role in domestic pollution in industrialized countries is played not only and not so much by gaseous products originating from combustion but by passive smoking to which about half of European children are allegedly exposed [43].

The combination of preventive measures includes also education about a healthy lifestyle, which includes eating and physical activity. In this context - more specifically a matter of the health sector - interdisciplinary collaboration and integration assume a great role: respiratory disease specialists interact with the Department of Prevention, regional health and social welfare services, and GPs.

### Secondary prevention of COPD (early diagnosis)

#### The rationale of early diagnosis

Given the epidemiologic values outlined above, of absolute importance, the National Health Service cannot act only passively (passive diagnosis) but must become a promoter of a series of actions in the field of primary and secondary prevention, and of rehabilitation.

The “2011-2013 scheme of the National Health Plan” [44] states that the management approach to chronic respiratory diseases that is capable of providing an optimal healthcare at a sustainable public costs includes “primary prevention and earliest possible diagnosis with standardized instruments, followed by rapid and appropriate treatments so as to prevent or delay invalidity, treating the chronic patients as much as possible in the community”. The document intends thus early diagnosis and not simply the promotion of interventions of information and education in the fight against the main causal agents (chief amongst which is smoking), or the activation of programs of reduction of occupational and environmental risk (about which we spoke earlier).

As already outlined, COPD is currently diagnosed at a late or even very advanced phase; in Spain it has been documented that only 60% of those who have chronic respiratory symptoms report to a doctor and only 45% of these are then referred for a spirometry [45]. The consequences of this situation are that, at diagnosis, the person’s physical performance is already largely compromised and the therapeutic interventions are less effective (see next paragraph). For this reason the National Health Plan stresses the need to arrive earlier at defining the presence (or absence) of the disease.

**Figure 3:**
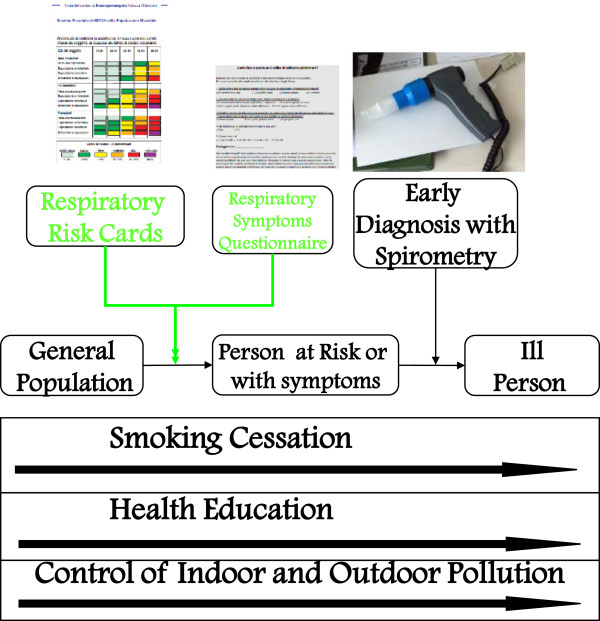
**Interventions for early diagnosis of COPD.** From [35] mod.

Early diagnosis (which should always be understood as a clinical, not just functional, diagnosis: see Figure 3) is considered important because it enables one to act immediately on the causes of disease (first of all, cigarette smoking) so as to impede or delay the effects of the progression of respiratory disease towards more severe and invalidating symptom levels [46,47].

It is known that only smoking cessation (apart from oxygen therapy in the advanced phases) can alter the natural course of COPD and improve survival in those affected [48]. In addition, by administering the most appropriate pharmacological treatments and - above all - commencing pulmonary rehabilitation in the initial phases of the disease it is possible to reduce exacerbations and improve the quality of life [46]. Initiating in this phase also interventions of education about the disease enables one to lighten the burden of stigma and depression that follow diagnosis. Since these psychological factors influence patients’ adherence to treatment as well as their quality of life the physician should know about them and know how to diagnose them [49].

If, then, secondary prevention offers these benefits, the next question to ask is who should undergo COPD screening: the general population? symptomatic individuals? subgroups at risk?

#### Population targets for screening

There is consensus that diagnosis should be based on screening of people *at risk* rather than screening the whole population. The latter would be hardly cost-effective given that the prevalence of COPD seems relatively modest in the general population whereas it is markedly elevated in smokers over 40 years of age [3]. Moreover, the system in Italy of hospital divisions of Pulmonology and Respiratory Pathophysiology as the locus for performing and analyzing the spirometry tests would in any case be insufficient to handle the great number of respiratory pathophysiology examinations needed to “screen*”* the entire population. Considering the global situation world-wide, also GARD/WHO does not recommend planning of spirometry tests for each single individual at risk [3].

It thus appears more suitable to diagnose unknown cases based on the recognition of symptoms that become manifest (i.e. a greater diagnostic suspicion) and/or through an evaluation of the level of risk that a person might have developed COPD when they present to the GP’s clinic for whatever motive (case-finding medicine) or, also, through public health initiatives. All these strategies, however, require greater awareness and sensitivity on the part of GPs and of primary care in general towards respiratory diseases, without forgetting that, according to the aforementioned study [45] a spirometry test was more often prescribed - and the difference was statistically significant (67.6 vs. 28.6%; p <0.001) - by a pulmonologist than by a GP.

**Figure 4:**
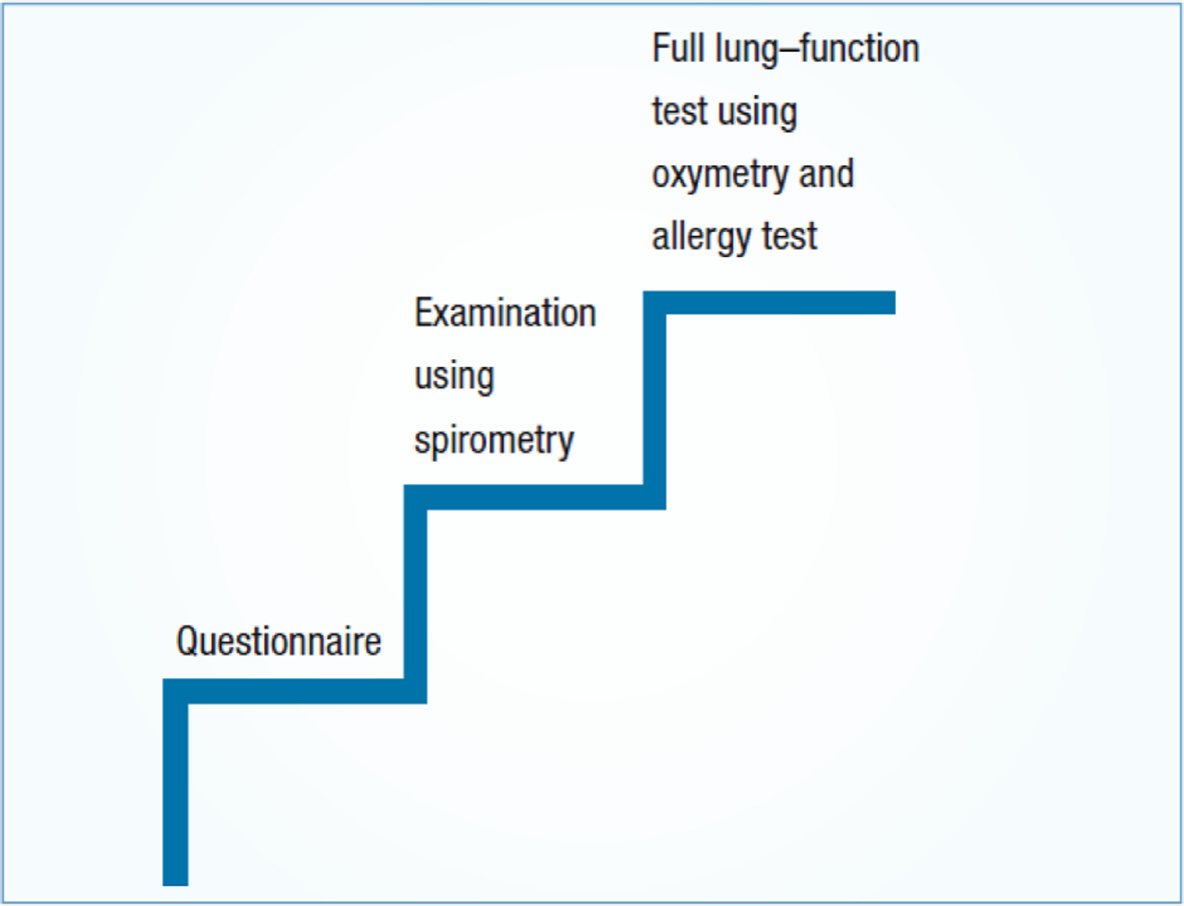
**A proposal for screening persons with COPD.** From [3].

Translated into practice this means that all symptomatic individuals aged over 40 years should undergo spirometry. The presence or not of respiratory symptoms will be checked/evaluated by the GP on the basis of the family history and lifestyle characteristics (risk profile) of the patient. It should be borne in mind that not always do patients consider their respiratory symptoms worth noting and of significance enough to report to the doctor [50,51] - hence the importance of a “case-finding*”* approach and of public health initiatives.Also self-completed questionnaires have been used for screening in the process indicated by GARD/WHO, for which they form the first “step” (Figure 4).

As shown above, the presence of respiratory symptoms often goes unnoticed or is not appreciated by patients who interpret their own symptoms as due to “physiological” aging or to their smoking habit. Consequently, they do not consult the doctor except in the case of episodes of worsening of the symptoms *per se* due to an exacerbation. More or less often, in such cases, also the GP underestimates the situation, diagnosing the episode simply as an acute expression and not as an epiphenomenon of an unrecognized chronic condition, omitting to examine in more depth the clinical picture. The net result, as we earlier pointed out, is that up to 80% of people with obstruction at spirometry according to GOLD criteria were without any diagnosis and, furthermore, less than half of those with severe obstruction had been earlier diagnosed [18].

The use of questionnaires with specifically targeted questions can help throw light on this shadow area, making patients aware of the need to investigate more deeply their own condition. In this sense, also initiatives of awareness raising addressed at the general population and/or at specific sub populations can help (see below). There is general agreement that questionnaires are useful and also about the questions that should be contained in them: age, smoking habit and the duration of smoking (expressed in pack/years, i.e. number of cigarettes smoked daily multiplied by the years one has smoked and divided by 20), presence of cough, respiratory sounds, previous diagnosis of asthma or COPD, and possibly additional questions related to body mass index and to the use of respiratory drugs in the past two years [52-54]. Also the collection of administrative information about the use of healthcare services in the past through a specific algorithm can be a useful screening tool for COPD, endowed with sufficient positive predictive value to be able to identify individuals at risk of having an undiagnosed COPD [55]. Instead of the need to have structured questions put by the doctor or professional nurse or by the healthcare operator during the patient interview, questionnaires are preferable in that they can be self-completed, also at a distance, and eventually completed/integrated in a subsequent check by the nurse or technician.

There are numerous possible questionnaires that can be used, followed, in the screened patients, by a simple spirometry test. Some authors propose, as an alternative to spirometry, to measure the PEF on the basis of some scientific evidence [52,53,56,57]: this measure, however, is controversial as a diagnostic tool. Some authors [58] have in fact shown that variations of 1% in forced expiratory volume in 1 sec (FEV_1_) are associated to variations (both in the normal population and in patients affected by bronchial obstruction) of 29% in PEF. This is due to the fact that among the determinants of PEF (elastic recoil, bronchial resistance and muscle strength) the contribution of muscle strength (including that of the facial and mouth muscles) is all the more preponderant the less compromised the other two factors are. All this weakens the use of PEF, above all in the earlier phases of disease in which the obstruction is probably not yet very marked.

In the following pages we report the Italian versions, modified with respect to the original, of two questionnaires that are easy to use, validated and used in epidemiological research on COPD. The first is the Respiratory Health Screening Questionnaire (RHSQ) (Table 2) for the screening of subjects at risk of COPD [53,56]. To calculate the risk of COPD, the score is interpreted as follows: < 16.5 points = low risk, 16.5 - 19.5 points = medium risk, > 19.5 points = high risk. The second questionnaire, much simpler than the first, is the COPD Screener™ (Table 3) which can be made available also online through internet [59], and it too has been validated [60].

**Table 2:** Respiratory Health Screening Questionnaire (RHSQ), modified

**Question**	**Answer**	**Score**
1. Age	40 -49	0
Age-group (years)	50-59	4
60–69	60-69	8
2. (a) Are you an active smoker?	No O	Yes O
(b) How many cigarettes do you smoke per day?	Cigarettes	
(c) For how many years have you been smoking?	Years	
3. Using the answers to question 2, calculate your pack/years		
*N.B. To calculate the pack years you have to do the following operation:*		
*number of cigarettes smoked per day x years you have smoked divided by 20.*		
	0-14	0
	15-24	2
	25-49	3
	50+	7
4. What is your weight?		kg
5. What is your height?		m
6. Using the answers to questions 4 and 5, calculate your body mass index:		
*N.B. To calculate the body mass index you have to do the following operation:*		
*Weight expressed in kg divided by height expressed in metres squared*		
Body mass index (BMI), kg/m2		
	> 29.7	0
	25.4-29.7	1
	< 25.4	5
6. If you have respiratory symptoms (cough, breathlessness) does the weather influence your symptoms?		
	Yes	3
	No	0
	I never have symptoms	0
7. Do you sometimes have a lot of phlegm even if you don’t have a cold or ‘flu?		
	Yes	3
	No	0
8. Do you ever cough up phlegm in the morning?		
	Yes	0
	No	4
9. Do you ever hear “whistling” sounds or other noises inside your chest when breathing?		
	Never	0
	Sometimes/often	4
10. Have you ever suffered from allergies?		
	Yes	0
	No	3

**Table 3:** COPD-population screener™ (mod)

**How can you understand if you suffer from pulmonary emphysema?**			
Mark with a cross the box of the answer that best describes the situation.			
The number beside the box serves to calculate the final score.			
1. In the last two weeks how many times did you have the sensation of being out of breath?			
***□*** *0 rarely or never*	***□*** *1 sometimes*	***□*** *2 almost always*	*□ 2 always*
2. Does it ever happen when you cough that you feel catarrh or mucus moves?			
***□*** *0 only occasionally when I have a cold or ‘flu’*	***□*** *1 a few days per month*		
***□*** *1 most days of the week*	***□*** *2 every day of the week*		
3. What is the answer to the following question that best describers your situation in the last 12 months:			
“I do less than I used to because I have breathing problems”			
***□*** *0 I don’t think so*	***□*** *1 this happens to me sometimes*	***□*** *2 it’s exactly like that*	
4. Have you smoked more than 100 cigarettes in your whole life?			
***□*** *0 no*		***□*** *2 yes*	
5. How old are you?			
*□ 0 from 35 to 49*	*□ 1 from 50 to 59*	***□*** *2 from 60 to 69*	***□*** *2 over 70*
**Total score**: ____________________			
**If the total is 5 or more** your breathing problems could be caused by a disease that is known as pulmonary emphysema or chronic bronchitis and that doctors call chronic obstructive pulmonary disease (COPD). Talk about it with your doctor, reporting the answers given to this questionnaire. Remember anyhow that any breathing problem, even banal but which lasts a few weeks or months, could be the signal of a lung disease; to exclude this it would be a good idea to talk about it with your GP.			

As an alternative, one can proceed sequentially starting from the identification of people at risk (these by unanimous consent are people over 40 or 50 years of age who are smokers, or ex-smokers since less than 10 years, or exposed to air pollution) administering as an initial step a questionnaire that helps to seek the respiratory symptoms and then submitting only those who result “positive” on the questionnaire to a simple spirometry test [3,61]. A further method is recruiting by invitation to specific people with certain characteristics to participate in the spirometry screening - the candidates for screening are thus self-selected [62]. This latter method, addressing smokers aged between 40 and 55 years, seems to improve the efficiency of the screening procedure, given that the frequency of disease carriers is particularly high.

The possibility of conducting a public health initiative with an “active call” to individuals at risk of COPD, along the same lines as already conducted in Italy for cardiovascular disease, will be illustrated in the next paragraph.

**Figure 5:**
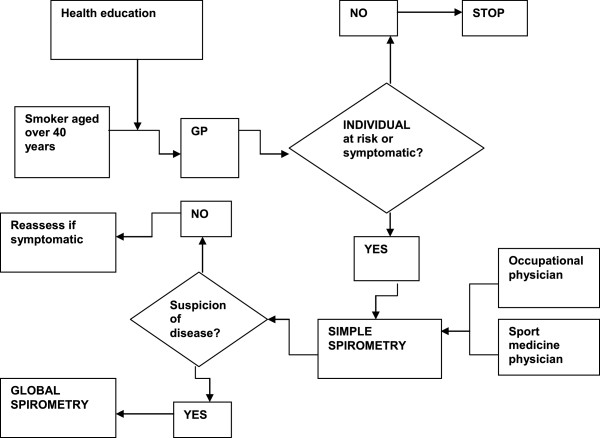
Flow chart for COPD diagnosis.

Summing up, the best approach is one of the sequences illustrated in the flow chart (Figure 5), in which two health professionals characteristic of the Italian situation are involved, i.e. the occupational health doctor and the sports doctor, whose role, parallel to that of the GP (Table 4), can be extremely precious in this phase of the diagnostic process of COPD.

**Table 4:** Process involving the GP

●	1a. The patient visits the GP because, through a health information campaign and also by means of a questionnaire, they have become aware of their own respiratory symptoms.
	Or:
●	1b. The patient goes to the GP for reasons other than the presence of respiratory symptoms, and it is the doctor who seeks for the presence of respiratory symptoms and traces the patient’s risk profile.
●	2. The doctor confirms or disconfirms the clinical suspicion of COPD.
●	3. In the affirmative, he will get the patient to perform a spirometry test.
●	4. If the spirometry shows an impairment, it requires confirmation through a global spirometry or a specialist referral.
	Nonetheless, people who result negative on the simple spirometry test but in the opinion of the GP are still suspect of chronic respiratory disease should be referred for further respiratory function tests, since simple spirometry is a test of low sensitivity.

##### Role of the GP

The use by the GP of the above-mentioned risk cards [63], available online [64], and/or of the questionnaire would be easy to apply. Inserting them within the online healthcare files in use would make it extremely easy to identify subjects at risk. Such activity (Table 5) could be carried out by the nursing staff present in group practices, community primary care units, healthcare homes, healthcare co-operatives, and all those forms of collaborative association that offer the possibility of using such personnel.

**Table 5:** Actions for the GP to perform

●	1) Indicate on the clinical record the status of smoker, ex-smoker, never smoker;
●	2) indicate on the clinical record occupational risk factors (e.g. welding, zinc workers, etc.);
●	3) train oneself and nursing staff in the use of risk cards, questionnaires, simple spirometry, and how to perform one;
●	4) use the computerized risk card;
●	5) administer the questionnaire to subjects at risk;
●	6) perform a simple spirometry (see below) for initial identification of disease;
●	7) perform a global spirometry on subjects with altered spirometry;
●	8) refer patients who present alterations to the pulmonary specialist for diagnostic confirmation, staging, and therapy prescription;
●	9) monitor subjects at risk who are still without manifest disease, and try to enroll them in a smoking cessation project;
●	10) refer to the pulmonary specialist subjects with negative results on spirometry but persistent diagnostic suspicion of chronic respiratory disease.
	The GP should be assisted by:
	- a secretary (point 1, 2);
	- a nurse under the GP’s supervision (point 3, 4, 5, 6, 7).

##### Role of the Sports/Occupational health doctor

An analogous path to that of the GP outlined in point 1b is suggested also for the other two health professionals involved, who have amongst their tasks to evaluate people’s respiratory function: the Occupational health physician and the Sports doctor (Table 6).

**Table 6:** Process involving the occupational health physician/sports doctor

●	1. In their activity the occupational health or the sports doctor performs a simple spirometry test (this is expressly required by law) or identifies a patient with respiratory symptoms and, based on the diagnostic suspicion, carries out “on their own” a simple spirometry.
●	2. A spirometry that reveals an alteration requires confirmation through a comprehensive spirometry or specialist evaluation.
●	3. Simple spirometry that does not show alterations in a symptomatic patient requires further verifications.

Even if, in all these cases, the GP is the key figure [65] who can successfully accomplish these actions, the GP must be assured a full coordination between the different players involved (patients, health workers, and health service structures) through the creation of a network that ensures “continuing” management of the patient (and not of the disease or of its single phases) and is centered on the patient.

In fact the models that attempt to re-design the care for chronic diseases identify for GPs a series of fundamental changes: from improving the possibility of access to the GP’s office to making available a clinical computerized support, to the possibility of obtaining instant help in clinical decision making (according to the “point of care” logic) - also with telemedicine, to support for the patient’s self-management. Nonetheless, alongside the GP, and often preceding the GP’s own action, there can be “moments of contact” with the national health service by the patient affected by COPD but not yet diagnosed, that should be capitalized on. We are referring here, besides the contact with Occupational health physicians and Sport doctors already mentioned, to use of Emergency services for acute respiratory problems. If certain specific characteristics are present, the emergency care doctor can - when the acute event is resolved and the person is discharged home - address the patient in case of suspicion to a follow - up pulmonary specialist evaluation.

#### Role of public health initiatives: active prevention

Among the array of possible initiatives, besides the individual intervention of the GP and Occupational health doctor, other healthcare professionals can collaborate with these two professional figures, integrating their activities. In public health initiatives, the effective instrument of prevention, once the target population to address has been identified, is that of the active call. Active prevention represents an articulated group of interventions, “actively” offered to the population at risk of COPD. The various subjects of the National Health Service involved in activities of primary and secondary prevention are involved in it in an integrated and coordinated way. Besides these, active prevention presupposes the conscious adhesion on the part of the citizen, whose will to participate in the screening programs and successive initiatives of prevention is requested. The active call has the advantage of soliciting interested subjects so as to implement the necessary actions and optimize the interventions of diagnosis and care.

In Italy some models have already been experimented and are in the course of experimentation - although not in respiratory medicine - and can have as their objective both early diagnosis (e.g. the campaigns of screening of bowel cancer) and true primary prevention itself. A good model for intervention based on the use of risk cards could be that proposed by a study begun in 2009 in the Veneto Region regarding cardiovascular disease which is about to be published [66]. The organizational model proposed is managed by the Departments of Prevention and has as its target the proactive assessment (through active call) of healthy individuals aged between 45 and 59 years. The Screening Center (SC) of the Department of Prevention operates in collaboration with the local health district and GPs: the SC prepares the lists of residents and submits them to the GPs, who help in the selection of subjects to contact, excluding those among their patients who bear any of the causes for exclusion (i.e. those who, on the basis of their records, carry a diagnosis of neoplasia in the active phase, progressive or severe neurological disease, cognitive decline or psychiatric disease, and chronic kidney failure). The GP should also exclude any persons who result to the GP already under treatment for ischemic heart or cerebral disease, arterial hypertension or diabetes mellitus with therapeutic indication. Citizens thus identified are invited (“active call”) to an appointment by letter with an eventual reminder in the case of non-respondents. Computer management is provided by a specific software program. To those who adhere, a healthcare assistant (HA) administers a standardized questionnaire and records data such as weight, height, body mass index (BMI), waist circumference, arterial blood pressure, and fasting glycemia. Based on the results of the interview and the individual’s conditions the HA proceeds to give the motivating reasons and the proposal for targeted preventive interventions differentiated according to the person’s classification in one of 4 categories (i.e. healthy without risk factors, healthy with risk factors, with abnormal parameters, subjects with pre-existing criteria of exclusion but not excluded due to error).

**Figure 6:**
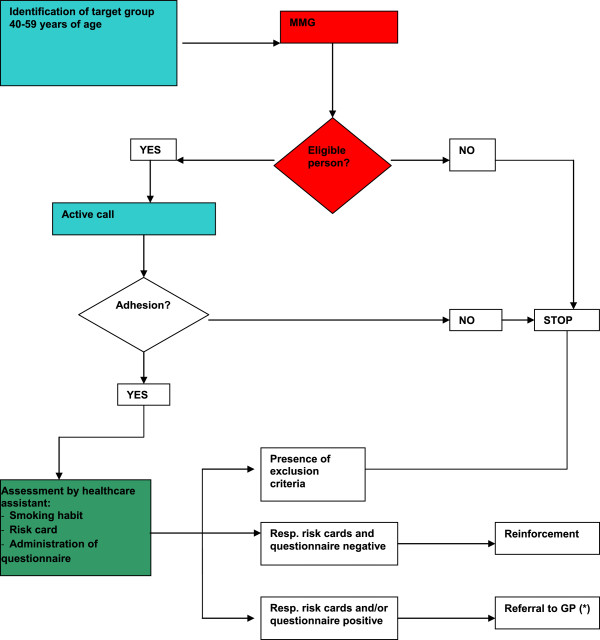
**Proposed model of respiratory disease prevention by active prevention.** From [66] mod. Department of Prevention; District; GP; (*) for clinical assessment and spirometry.

A similar model (which as can be seen can combine primary and secondary prevention) could, with the appropriate modifications, be proposed also for primary and secondary prevention of COPD and would thus give the community two possibilities of screening: the case-finding approach on the part of the GP, and “active call”.On these premises the following flow chart has been created (Figure 6).

#### Screening procedures with respiratory function measures

The above model presupposes an increased availability of spirometry tests with respect to the present and to achieve this there are diverse options:

1. Spirometry performed at the GP’s office, directly by the doctor or the nurse, gathering periodically all the selected patients on dedicated and programmed (or programmable) days (and hours).

2. Spirometry performed at the GP’s office, by technical personnel made available by the department of Pulmonology or Respiratory Pathophysiology at periodic intervals.

3. Spirometry performed at a Pharmacy by specially trained personnel [67].

4. Spirometry performed at a facility of the local social health services - which is usually “closer” and more accessible to the population than the hospital. In such cases and always with planning of the appointments, outpatient specialists or trained nursing staff linked to the outpatient clinic (when available) can be requested to perform the spirometry test. If not available, the feasibility of carrying out the test by technical staff made available by the Pulmonology or Respiratory Pathophysiology Unit can be evaluated.

For each of these approaches it is also possible to have the diagnosis made or a comment given by the reference specialist, possibly (where available) through telemedicine. The professional figure who carries out the test and who assesses the result, in contrast to other procedures, is of great importance, given that the test, to be significant, must be carried out and recorded “in the correct way”. In other words, whoever performs and, in any case, records the result of the test must have adequate training and experience, given that the diagnostic significance of spirometry is both patient- and operator-dependent, with full collaboration required between the two figures involved [68]. Regular calibration of the spirometer is essential and should be done on a regular basis according to the manufacturer’s indications.

The most frequent error in the phase of execution is that of a forced expiratory phase which is too-short (this occurs often in severely obstructed patients) and interrupted early. This error can be detected only if one can visualize in real time the expiratory curve, which therefore needs to be visible in the spirometer used for the screening. To avoid the error of a too brief expiration, an alternative can be to assess the FEV_1_/FEV_6 _ratio, especially for COPD screening programs in high risk populations [69]. The fact that the spirometer contains the predicted values enables one to make an immediate judgment. If the device is able to print out immediately a report or if it is connectable to a printer make the service complete and enables the results of the test to be saved.

#### Available equipment

Different types of portable spirometers exist that can be used and managed in outpatient clinics not dedicated to respiratory pathophysiology. The element for measuring (transducer) can be a turbine or a pneumotachograph. Whatever device one wishes to use, for reliable results the device must not only be able to memorize the data collected (for purposes of making a report - also at a distance - and storing data) but also to show in real time on the display both the numerical values and, above all, the curves generated by the patient, in order to allow an assessment of the reliability and reproducibility of the test. In alternative, the spirometer must possess these characteristics when interfaced with a computer. Being able to view in real time the curves enables, as we said, to detect immediately the size of the effort made by the patient and other errors in the performance of the test.

The performance of these portable spirometers has been compared (particularly in the pediatric setting) with conventional spirometers. Comparing three groups of children (with clinical asthma, suspected asthma, and healthy controls) no significant differences were found between the two categories of spirometer [70]. A more recent study has verified the accuracy and precision of these spirometers in the general practice setting [71]: 80% of the devices were turbine spirometers and 16% pneumotachograph spirometers. In general all devices showed a mild overestimation (mean 25 mL) both of FEV_1_ and forced vital capacity (FVC)^(a)^, but some of them showed substantial deviations. Significantly greater deviations with respect to the turbine spirometers were observed in the pneumotachograph type concerning FEV_1_ and FVC. Finally, the long term reliability of turbine spirometers has been demonstrated by a 3-year study carried out in Denmark [72].

In general, the values measured by portable spirometers although repeatable should not be considered as interchangeable with those obtained by conventional spirometers [73], so their use is best as a first step in diagnosing obstruction and to monitor its evolution in time. Naturally, the technical quality of the device does not guarantee the quality of the execution of the test given that, as we said, the periodic calibration, and constant adjustment based on pressure, temperature and humidity, plus experience in performing the test are all equally influential on the success of the test and its reliability [68].

The health operator must know how to instruct the patient both with words and “body language” (spirometry is a difficult test also for the patient, especially if ill); in particular the instruction must cover (and continue throughout the execution of the test) both the initial phase of the forced maneuver (peak flow) and the “holding” of the expiratory phase for at least 6 seconds, before taking in another deep breath. For this, points of “bio-feedback” can be useful to let the patient see the curves while they are breathing [74], also through incentives with images simultaneous to the execution of the forced maneuver.

Not by chance the aforementioned Dutch study on GP offices showed deficiencies in the calibration [71] and another study, also in the field of general medicine [75], showed the existence of serious problems concerning the training of health operators, the frequency of the calibrations, as well as the effective use of the device (45% of those who had a spirometer did not use it). Finally, a problem that should not be overlooked is the risk of crossed infections associated to the use of turbine or pneumotachograph spirometers. These can be prevented by use of disposable filters [76]. Also disposable transducers have recently been put on the market.

From what has been said above, it appears evident that carrying out spirometry in the GP setting is a burdensome task in terms of the human and economic resources and time involved. Confirmation of this, in Italy, comes from a large study carried out a few years ago (on symptomatic patients only) that showed that spirometry is certainly performable in the general medicine setting, but GPs are not very enthusiastic about it (mainly because of lack of time) and it is further complicated by non-negligible problems regarding the maintenance of adequate standards of performance and interpretation [77].

While not denying that an associated GP clinic or a GP with particular specific interest can offer such a service (possible especially if the technical personnel comes from or has been instructed in a specialized hospital department), this latter does not appear currently feasible for the average Italian context, also because of the time required for performance of the spirometry, a burden difficult to support for general medicine that is not strongly organized, considering the population group which theoretically needs such a test.

This consideration is not limited only to the Italian situation. Enright, well-known American respiratory pathophysiologist, in fact showed that it is better not to do spirometries at all in the primary care setting rather than do them incorrectly [78] and in an editorial accompanying a study on spirometries performed by local community nurses he concluded that it is recommendable to provide GPs with good quality spirometries rather than spirometers [79].

#### How to define bronchial obstruction

Spirometry - regardless of the different techniques - measures the volumes and flows developed by the patient while he/she breathes into the device. The test consists generally of two maneuvers, a slow one and a forced one: in both cases the patient breathes to the maximum capacity (inspiratory and expiratory), but in the first case in a slow mode and in the second case with as intense an expiratory force as possible. For a correct analysis of the forced maneuvers it is necessary to have at least 3 reproducible trials, utilizing for the subsequent analysis the one with the highest sum of FVC and FEV_1_. The results are compared with theoretic, or predicted, values gathered from healthy subjects who are presumed to represent the normal population of reference. The theoretic values should be periodically updated, however, in that the rapid improvement of social, physical and nutritional conditions can lead to variations of the reference values. It is known, moreover, that one of the determining factors for the calculation of predicted values is race (besides age, height and gender), and the mixture of races typical of the present era is further cause for caution in using predicted values. The comparison is thus expressed as a percentage of the measured to theoretic value and the presence of alterations is defined by measured values below the normal values.

To assess bronchial obstruction a measured value in the forced expiration (i.e. the maximum expiratory volume measured at the first second from the start of expiration, FEV_1_) is correlated to the measure of the vital capacity (i.e. to the total volume that can be mobilized by the person (vital capacity, VC^(b)^, or forced vital capacity, FVC). In the presence of obstruction (or in cases of doubt) the measure must be repeated “after” the administration of a bronchodilator (usually salbutamol 400 mcg, in the absence of contraindications): the persistence of an altered value (see below) or its incomplete normalization defines the obstruction characteristic of COPD. If even after the bronchodilator doubt should persist this is an indication for the execution of a global spirometry, which include the measure of residual volume (that should also be performed in all with proven obstruction for a better functional characterization of the obstruction: see below).

There exist two criteria for defining the presence of bronchial obstruction: the so-called fixed ratio and the lower limit of normality (LLN). In the first case an obstruction is considered to be present if the ratio FEV_1_/VC is lower than 70%. In the second case, the presence of obstruction is diagnosed if the measured value is lower than the 5^th^ percentile of a healthy population composed of non-smokers, or below 88% in males and 89% in women.

There is now as much agreement among respiratory pathophysiologists that the definition based on the fixed ratio is inaccurate as there is agreement among pulmonary specialists working in the field about continuing to use it: more than 86% of 29 units of pulmonology in a recent study [80]. The main argument of the former is that it can generate underdiagnosis in young people (aged under 40 years of age in women and under 50 in men) and overdiagnosis in the elderly [81], in practice misclassifying obstruction and generating false positives in the more advanced age classes (given that respiratory function declines with age, such as to justify in itself a reduction of the FEV_1_/FVC index). This diagnostic criterion for obstruction is suggested also by the recommendations contained in a joint ERS-ATS statement on the interpretation of spirometry [82].

The fixed ratio is instead recommended by the GOLD [83] and AGE.NA.S [36] guidelines, inasmuch as there exist arguments also in favor of use of this latter criterion. The first is the AGE.NA.S acknowledgment that *“*reliable estimates of distribution of the values of the FEV_1_/FVC ratio in the various age groups do not exist and that thus a diagnosis based on values below the 5^th^ centile of distribution of FEV_1_/FVC in the reference population would be impracticable*”.* Secondly, the excess of prevalence that one observes with the fixed ratio in the elderly is not the same for all classes of severity of obstruction, there being in GOLD class II a greater correlation to the clinical diagnosis with the fixed ratio than with the LLN [84]. In addition, the disadvantage derived from an excess of prevalence described (less specificity) by the fixed ratio may be counter-balanced by the advantage of its greater sensitivity, at least in some age-classes. In fact, those who are diagnosed as obstructed with the fixed ratio have more hospital admissions and a higher rate of mortality than those who are diagnosed with the LLN; in other words, it could be that the LLN underestimates COPD (which is not solely bronchial obstruction - see section How to organize treatment: defining the disease severity) [85].

Finally, given that the diagnosis of COPD, particularly in the elderly, is never only spirometry-based, but rather on the contrary diagnostic suspicion (presence of cough, sputum, dyspnea and history of smoking or exposure to environmental risks) precedes the spirometry, in the opinion of pulmonologists “working in the field” and also of our working group the choice can be left to the specialist which of the two criteria of obstruction to use, e.g. with which one he has more familiarity, knowing that the definition of obstruction can still easily be based on the fixed limit of 70% in the ratio FEV_1_/FVC, given that, as evidenced by the AGE.NA.S guidelines, “the potential diagnostic error, due to the choice of the value 0.70 as the sole lower limit of normality of the FEV_1_/FVC ratio, will be reduced by the dimension of clinical probability of disease before the execution of the spirometry test”. This, for so long as the still missing epidemiologic data are not available.

In practice for the clinician it is thus sufficient to know that the use of the fixed ratio determines an area of uncertainty due to the potential cases of under- and overdiagnosis, respectively, in young people and in the elderly. He will thus consider alternative, complementary diagnoses or investigations (or will refer the patient to a specialist for evaluation) in elderly with a fixed ratio below 70% but without typical symptoms of COPD and in young people with typical symptoms but with a ratio above 70% [86].

Finally, besides writing a correct medical report on the examination, the specialist will need to make an effort to write the report in a way that is “legible” and comprehensible to the GP: the respiratory scientific societies and those of general medicine should jointly undertake an initiative to standardize the medical report.

### What to do after early diagnosis

#### How to organize treatment: defining the disease severity

**Figure 7:**
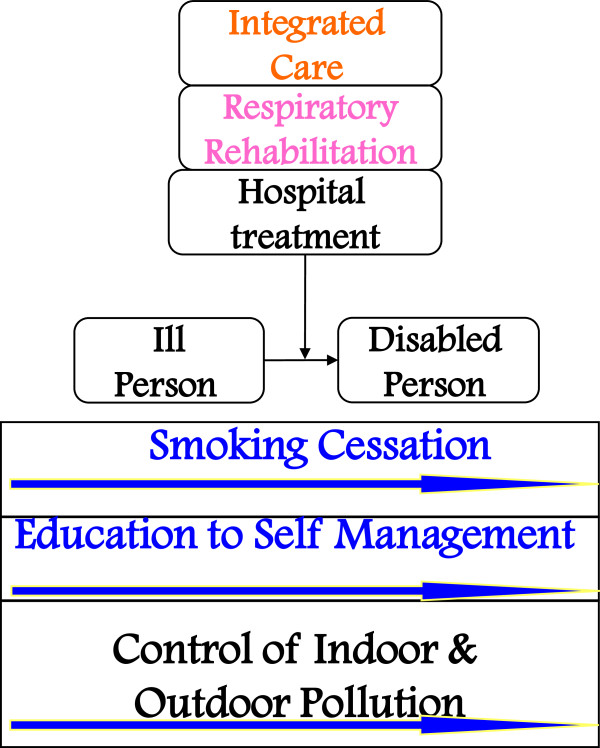
Interventions for blocking the progression of the disease.

Once the presence of obstruction has been diagnosed with simple spirometry, a person with suspected COPD should be referred to functional respiratory unit for diagnostic confirmation that includes global spirometry with reversibility testing. Referral should aim at confirming the diagnosis of obstruction as well as at staging its severity so that he/she can be inserted in the most appropriate therapeutic course (see sections How to organize treatment: defining the disease severity, How to organize treatment: educating patients about their disease, How to organize treatment: smoking cessation and related pharmaceutical drugs, How to organize treatment: use of respiratory drugs). Once diagnosis has been confirmed and the level of severity established, the course of treatment will consist always in smoking cessation, pulmonary rehabilitation (in practice, training in exercise tolerance), reduction of exposure to pollutants, pharmacological therapy correlated to the disease severity (not simply to the severity of obstruction - see below), prevention and treatment of exacerbations, this latter both at home and in hospital. Accompanying these measures, of not less importance is education about the disease^(c)^ which makes the patient a conscious and co-responsible partner of the treatment (Figure 7).

The aim of early diagnosis is to detect patients who are affected by COPD (without knowing) at the earliest possible stage, if possible when still at a mild or moderate functional stage of obstruction. At such stages, in fact, the cited interventions (smoking cessation, rehabilitation, pharmacological therapy, etc.) can significantly reduce the impact of respiratory function decline and exacerbations, and improve the quality of life. We repeat that early diagnosis must be followed by a staging of the severity and by the therapy that is most appropriate. It is worth repeating here also that one must stage and treat the patient considering the severity of the disease and not that of bronchial obstruction: it has been demonstrated in fact that bronchial obstruction is not the only index of severity that correlates with survival [87] and that equivalent levels of obstruction can correspond to very different levels of invalidity and prognosis [88]. This is because disease severity appears to be determined not only by the impairment of pulmonary function *per se* but also by other characteristics such as symptoms, tendency for exacerbations, nutritional status and presence of comorbidities.

The specialist will thus have not only the task of confirming the spirometry diagnosis of obstruction but also of classifying its degree of severity in reference to the GOLD, ERS-ATS and AGE.NA.S guidelines, according to the scheme in Table 7.

**Table 7:** Staging the bronchial obstruction

**Severity**	**FEV**_**1**_**/FVC after bronchodilatation**	**%predicted FEV**_**1**_
MILD	< 70%	> = 80%
MODERATE	< 70%	50-80%
SEVERE	< 70%	30-50%
VERY SEVERE	< 70%	< 30%

The specialist will need to assess in the COPD patient: i) the level of dyspnea (diverse scales exist for quantifying dyspnea, but the one most widely used – also in Italy [80]- is the Medical Research Council (MRC) scale [87]) (Table 8), ii) body mass index (BMI), i.e. weight in kilograms divided by height expressed in meters, squared, and iii) exercise tolerance, measured with the 6-minute walking test (6MWT).

**Table 8:** MRC dyspnea scale

**Level of dyspnea**	**Symptoms referred or reported**
0	Presents fatigue after strenuous exercise
1	Averts shortness of breath when walking fast or walking uphill
2	Walks more slowly than someone of similar age due to dyspnea
3	Stops after a few minutes or 100 meters when walking
4	Obliged to not move from home; dyspnea when getting dressed or undressed

The MRC scale, together with BMI, 6MWT (exercise tolerance), and FEV_1_ (bronchial obstruction) in a combined index known as the BODE index provides the most complete photograph of the condition of a person affected by COPD (Table 9), and can give a more accurate picture of the patient’s prognosis than FEV_1_ alone.

**Table 9:** The BODE index

**Score (from **[91]	**0**	**1**	**2**	**3**
FEV_1_ (%pred.)	≥ 65%	50-64%	36-49%	≤ 35%
Distance walked in 6 mins (m)	≥ 350	250-349	150-249	≤ 149 m
Level of dyspnea (MRC)	0-1	2	3	4
Body mass index (BMI)	≥ 21	≤ 21		

Besides these criteria, in addition to spirometry, also information about the frequency of episodes of exacerbation helps to define the severity of the disease [89] as well as modifying substantially the impact on the healthcare costs. It appears worthwhile to recall that some of the cited indices - in particular the MRC scale - could easily be used also by GPs in their clinical practice.

In summary, to be able to correctly evaluate the treatment outcomes that truly count for persons with COPD [90] one must pass from an assessment of spirometry alone to an assessment that takes into account all the above dimensions (Table 10).

**Table 10:** Global evaluation of the patient affected by COPD

**Dimensions to take into account when defining COPD severity and verifying treatment efficacy**	
●	Respiratory function
●	Quality and intensity of symptoms
●	Frequency and severity of exacerbations
●	Functional status of the patient
●	Therapies: need/adherence
●	Satisfaction and quality of life

Hence - in practice - one has to pass from the established traditional scheme (Table 11 see (A. Current approach to COPD**)**) to a new scheme (Table 11 see (B. Desirable approach to COPD)) which clearly shows, together with what has been said above, that the key to obtaining results cannot but be through integration between the different health services and, hence, a reappraisal of the respective roles of the hospital and local community services.

**Table 11:** Diagnostic suspicion

**A. Current approach to COPD**	**B. Desirable approach to COPD**
Diagnostic suspect	Diagnostic suspect
↓	↓
Spirometry	Spirometry, Multi-dimensional evaluation
↓	↓
Pharmacological therapy	Integrated treatment and Rehabilitation
↓	↓
Spirometric follow-up	Multi-dimensional follow-up

Treatment is composed of non-pharmacological therapy and pharmacological therapy that are well described and analyzed in the AGE.NA.S document and in the joint societies statement on the integrated management of COPD [35], and will be outlined in more detail in paragraph 3.3 and relative subparagraphs.

This global treatment can improve pulmonary function and quality of life and reduce exacerbations, as long as all the phases of care are managed in a coordinated and integrated mode, and the person affected by the disease knowingly collaborates, i.e. is educated about the disease.

The programmed follow up, i.e. visits aside from those requested by patients themselves on account of clinical variations arising, will have a frequency that differs according to the severity of the disease. The suggested schedule of these visits is reported in Table 12, taken from the joint society document [35].

**Table 12:** Recommendations for follow up of patients based on their clinical conditions

**Activities**	**Follow up of chronic bronchitis (without obstruction) and of mild (obstruction + FEV**_**1**_ **> 80%) asymptomatic COPD**	**Follow up of COPD with obstruction + FEV**_**1**_ **> 80%, exercise dyspnea and comorbidities**	**Follow up of COPD with obstruction + FEV**_**1**_ **< 60%, exercise dyspnea, frequent exacerbations and comorbidities**	**Follow up of COPD with obstruction + FEV**_**1**_ **< 50%, respiratory failure and comorbidities**
	**Every two years**	**Once a year**	**Once a year**	**Once a year**
Smoking cessation, if smoker	All and/or anti-smoking center	All and/or anti-smoking center	All and/or anti-smoking center	All and/or anti-smoking center
Clinical assessment (including dyspnea index, BMI, with eventual use of questionnaires) and of risk factors	GP, specialist	GP, specialist	GP, specialist	GP, specialist
Pulse oximetry	GP, specialist	GP, specialist	GP, specialist	GP, specialist
Simple spirometry	GP, specialist	GP, specialist	GP, specialist	GP, specialist
Pulmonologist consultation	Pulmonary specialist in the case of diagnostic doubt	Pulmonary specialist	Pulmonary specialist	Pulmonary specialist

From [35].

#### How to organize treatment: educating patients about their disease

As specified earlier, patient education about the disease plays a key role in the management of chronic diseases, and has the aim to make patients themselves an ally in the care and treatment plan, seeking to change their role from that of a passive object of the health intervention to the subject on which this latter is focused. It includes diverse components, aimed at giving the person essential information about the characteristics of their disease, increasing their motivation and understanding in order to modify the behaviors, transmitting skills, and providing support for the emotive aspects of symptoms. These interventions all together are able to increase the individual’s capacity of self-management and to guide them to make the best use of the resources available from the national health system.

Each intervention must also be adapted to the cultural level (*literacy*) of the person receiving the intervention: people belonging to different ethnic groups and religions and/or with different levels of education will benefit from different educational interventions.

Self-management is not only of benefit for the patient and patient’s family but seems also effective in reducing hospitalizations without side effects or adverse events occurring [91]; but while for other chronic diseases such as diabetes there has been significant progress in education about the disease, especially in Italy, for COPD there is still a long road ahead [92].

In experiences conducted abroad that have yielded good results, besides face to face teaching sessions or “lessons” conducted by trained personnel, elements of management planning (similar to those already available - and well known - for asthma) have been made available both in the stable phase and in the case of worsening of symptoms, as well as telephone contacts made periodically by the reference person responsible for the care and/or on request by the patient [93]. The availability of “action plans” alone that are not integrated into the global education about the disease can improve the capacity to recognize early exacerbations, but does not seem able to modify either the use of health care resources or the quality of life [94].

Still in the context of disease education, i.e. as part of the overall “package”, the availability of tele-assistance can also play a crucial role [35]. By this term is intended the use of technologies that go beyond the telephone (made available for bi-directional contacts) and include also telemonitoring (which today makes possible control at a distance of spirometry, pulse oximetry, walked distance and symptoms), telecameras and internet. Such exchange of information finds its chief indication in the variations of clinical stability (such as are perceived by the patient) and allows the doctor to take into account the vital parameters of their own patient before they decide to seek hospitalization or emergency care [95]. Today we know that the use of these instruments makes it possible not only to reduce the “urgent” recourse to the national healthcare system but also to improve quality of life [96].

Care of the psycho-social aspects of the disease is another aspect of disease education: lack of breath induces the patient to a reduction of physical exercise and autonomy, with consequent anxiety and depression. There is not strong evidence that interventions of this type substantially influence the use of healthcare resources and quality of life, but nevertheless the studies carried out on asthma recommend their use [97].

Smoking cessation is able to modify the natural history of the disease and, together with pharmacological therapy and pulmonary rehabilitation, help to improve symptoms, exercise tolerance and, as a result, quality of life. However, in order to gain the benefit of these treatments, the patient’s adherence to what is prescribed is of obvious importance. In COPD it has been demonstrated that such adherence is very low. The reasons underlying this poor adherence are multiple, and are linked to patients and their motivation, to the doctors that prescribe the treatment and their capacity to be on a “wavelength” with the patients, to characteristics of the treatment itself, in particular for treatment with inhaler devices. For these latter the challenge is not only to develop devices that make the administration as practical as possible (as has been done for insulin therapy) but also to simplify the treatment plan, given that the more complex the therapy is, the greater the risk of lack of compliance, also in view of the fact that the patients concerned are often elderly, affected by chronic comorbidities and receiving different therapies, often prescribed by specialists who are not aware of the overall treatment panorama [98].

There are diverse approaches to resolve the problem of a correct and constant use of inhaled drugs: facility of use of the inhaler device, knowledge about the patient’s preferences, the cost of the drugs and of their prescription (in Italy people affected by COPD are not exempt from healthcare costs for the disease), steadfast and patient teaching on inhalation techniques by the personnel, the possibility of monitoring the person’s effective capacity in using the inhaler - only to cite the most important, and they need to be applied all together at the same time [99].

The use of generic drugs and the substitution of the drugs prescribed by the doctor with others proposed by the pharmacist can be a source of confusion for the patient, especially if he/she has been educated to use one inhaler device while the drug that the pharmacist actually gives employs another device.

There is no doubt, thus, that attention to the education of people affected by disease is an integral part of the overall management that should be implemented, and that such implementation should be without any elements that are not controllable or a source of confusion.

Patient Associations could play an important role in this process: they can help associates (both patients and relatives or caregivers) in the educational process not only by producing booklets or other printed **(**or online) material but also organizing groups for self - help. Patient Associations could also seek to influence politicians on the problems encountered by patients and caregivers in the management of the disease.

#### How to organize treatment: smoking cessation and related pharmaceutical drugs

It is well established that smoking cessation slows down the progression of COPD towards more severe levels of disease and invalidity (this is due also to its effect on the frequent smoking-related comorbidities) [36,83]. In the context of COPD smoking cessation is an essential therapeutic measure and as such it must be understood, above all by the doctor that has charge of the patient; putting it in another way, smoking cessation should be the first provision made at the moment of diagnosis of COPD in an active smoker [36,100].

A minimal intervention of the doctor, known also as the method of the 5 “As” (Table 13) [36] to set a person on the course of smoking cessation should be carried out in all patients who are smokers, independently of whether they are affected or not by COPD, and it can be implemented with success by nursing staff. If disease is present, the doctor will add to the usual message (i.e. regarding how cessation can prevent the insurgence of smoking-related diseases) information about the strong therapeutic reasons that make smoking cessation a ‘must’ and using the respiratory function tests (principally spirometry and blood gases analysis) as a motivational lever. It has in fact been demonstrated that combining the use of spirometry with help in the attempt to quit smoking yields higher percentages of abstinence than not using the respiratory function test [101,102].

**Table 13:** **Method of the *5 As *** **for smoking cessation**

●	**Ask**
	Ask about smoking status with open-ended questions
●	**Advise**
	Inform about short and long term damage caused by cigarette smoking as well as about the benefits of quitting
●	**Assess**
	Evaluate the patient’s motivation and willingness to quit
●	**Assist**
	Help the patient in the attempt to quit smoking
●	**Arrange**
	Plan checkups and the follow up
	Prevent relapse

From [36].

The desirability and necessity of a more intense intervention than the minimum are justified first of all by the fact that there exists a direct dose-response correlation between the intensity of the intervention (minutes of contact) and its efficacy expressed as the percentage of long term abstinence [103]) and also by the fact that cigarette smoking is capable of creating a very strong physical and psychological dependence. A person who continues smoking in the presence of symptoms and notwithstanding a diagnosis of smoking-correlated disease is considered as showing a high level of dependence. Thus solely the advice to quit (which is the most widespread approach used in Italy) may, in the majority of cases, not be sufficient, and also the minimum intervention, which in any case should be always implemented, may not be enough in persons who almost always have previously attempted on their own to quit but always relapsed. Thus, based on the person’s clinical conditions and smoking characteristics a specialized smoking cessation intervention should be implemented that in addition to pharmacological support (which should always be administered) can provide psychological-behavioral assistance [35,36,83].

This type of intervention –which obtains the maximum percentage of long-term abstinence – can be provided both directly by the pulmonologist, if adequately trained and flanked by a psychologist (also part-time), and by specialized centers [104] that can be contacted directly by the patient through the toll-free telephone number of the National Health Institute (anti-smoking toll-free number: 800 554088).

It is important to remember that smokers affected by respiratory disease urgently need to quit and consequently it is the specialist who must assume a proactive and continuative role, i.e. providing continuing assistance to smoker patients suffering from respiratory disease with regular assessment of their smoking status, use of pharmacological therapy and provision of behavioral support, including treatment in the habitual routine management of their respiratory disease. Pharmacological treatment is aimed at reducing symptoms of abstinence and the satisfaction derived from smoking, psycho-behavioral therapy at modifying those behaviors that constitute, in the patient’s life, equally potent stimuli to smoke [100].

In practice, the doctor should collect - and report in the medical record - specific information on the patient’s smoking history, measuring the pack/years (i.e. the number of cigarettes smoked daily divided by 20 and multiplied by the years of smoking habit). The doctor should immediately begin treatment to quit, independently of the patient’s age, and using first-line active drugs unless there are contraindications: these are the different forms of nicotine replacement therapy (NRT) which is available in numerous formulations, varenicline and bupropion (in slow-release formulation) for oral administration. The best mode for judging if the therapy is sufficient is the disappearance or containment of the symptoms of abstinence, in particular craving to smoke; in the case of persistence of craving the drug dose should be increased or accompanied by a diverse formulation (if NRT) or by an additional drug. Doctors who consider themselves not sufficiently trained for this type of assistance should refer the patient to a specialized outpatient clinic (information about which can be found in the annual register edited and updated yearly by the National Health Institute [104]).

Whatever the health professional who assists the patient, all persons affected by COPD should be assessed for smoking at least twice a year, better at each programmed checkup. The checkup should include a biological marker of smoking (expired CO, COHb or salivary cotinine). The prescription of drugs to quit smoking must be directly linked to the prescription of the respiratory drugs.

Patients who in no way intend to attempt quitting immediately and in sudden fashion can be proposed a “reduction to quit” [105,106], i.e. the use of pharmacological therapy (see above) while they are allowed to continue to smoke. The intermediate goal is to reduce, with the aid of therapy, the quantity of cigarettes smoked daily (to at least 50% of the usual amount in 4-6 weeks). The final goal is to quit completely within 6-9 months. With this mode, the most studied drug is NRT that, in the forms as needed (chewing gum, inhalers, tablets) can be used as a real alternative to the cigarette [107]. The treatment (which can also include a transdermal patch, depending on the patient’s preference and smoking habit characteristics) can be interrupted if the goal of 50% reduction of cigarettes smoked (and a parallel reduction of CO) is not obtained within six weeks. It can be continued if a lasting and significant reduction in the number of cigarettes smoked has been reached, but telling the patient that at this point the goal has changed to “reduction of damage”. If the treatment is successful definitive cessation can be attempted and, in the case of reaching abstinence, the therapy is continued for at least two or three months.

Since the development of lung cancer is more frequent in COPD subjects, the specialist can when advising to “reduce in order to quit” refer not only to the COPD therapy but also to the prevention of lung cancer [108]. Finally, there is currently no scientific evidence that the so-called electronic cigarette can be used with the aforementioned scope, in that these are non-standardized products that have not been sufficiently tested [109].

#### How to organize treatment: use of respiratory drugs

There is agreement among the various official documents of scientific societies or working groups that the degree of pharmacological therapy should be based on the level of severity of the patient, who, as explained above, should never be assessed only as regards the level of obstruction. Whatever the level of severity, and beginning right from mild stages there are common therapeutic measures.

Alongside the first and foremost important treatment, i.e. assistance in smoking cessation (if necessary implemented in a “intensive” mode by specialized facilities or health professionals) but in any case with the use of specifically active drugs, there should also be prescribed promotion of physical exercise and anti-influenza and anti-pneumococcal vaccinal protection. Short-acting bronchodilators “as needed” should also be recommended (see below = SAMAs or SABAs).

In situations of greater severity (including failed control of symptoms) beginning from the moderate stage long-acting bronchodilators (LABDs) should be prescribed, which exist in two families: the beta-2-agonists and the anti-muscarinics. Doctors can choose the drug that they consider most appropriate for the type of patient (individualized treatment) from among the two families, whose components are now more numerous than in the recent past.

The characteristics of each of them are listed in Table 14, taken from the joint society document [35]. One should bear in mind that the most recent drugs are backed up by fewer studies of efficacy and safety than the more consolidated drugs.

**Table 14:** Bronchodilators for the treatment of COPD

**Class**	**Drug**	**Characteristics**
Long-acting anticholinergics (LAMAs)	Tiotropium	Length of action 24 hours
	Glycopyrronium	Length of action 24 hours
	Aclidinium	Length of action 12 hours
Long-acting beta-2-agonists (LABAs)	Formoterol	Length of action 12 hours
	Salmeterol	Length of action 12 hours
	Indacaterol	Length of action 24 hours
Fixed inhaled corticosteroid (ICS)/ LABA combinations	Salmeterol + fluticasone	Length of action 12 hours
	Formoterol + budesonide	Length of action 12 hours
	Formoterol + fluticasone	Length of action 12 hours
Phosphodiesterase-4 inhibitors	Roflumilast	Oral - Length of action 24 hours
**Other bronchodilator drugs**		
Short-acting beta-2-agonists (SABAs)	Salbutamol, terbutaline, fenoterol	Rapid onsetof action. Length of action 4-6 hours
Short-acting anticholinergics(SABAs)	Ipratropium and oxitropium bromide	Rapid onset but slower than SABAs.
		Length of action 6-8 hours
Methylxanthines	Slow-release oral theophylline	

In still more severe cases (defined also by the presence of exercise dyspnea) two LABDs can be combined and associated, in the case of frequent exacerbations or asthma comorbidity, with an inhaled corticosteroid (ICS); in this subset of patients recent studies seem to have shown significant results also using dual bronchodilation with LABA and LAMA associated [110,111]. In stages of maximum severity (i.e. severe COPD, based on the above-outlined parameters) in patients with a history of frequent exacerbations, a phosphodiesterase-4 inhibitor can be added to the bronchodilator therapy. Theophylline warrants a special note: on account of its bland broncho-dilating action and due to the significant side effects associated with its use, the main evidence for its use is in a small percentage of patients, or - as recent evidence confirms - in patients with refractory COPD, to restore their sensitivity to steroids [112]. Apart from this indication, the use of theophylline is usually limited to use as an additional therapy in patients with more severe disease when the symptoms persist despite a correct use of other treatments.

**Figure 8:**
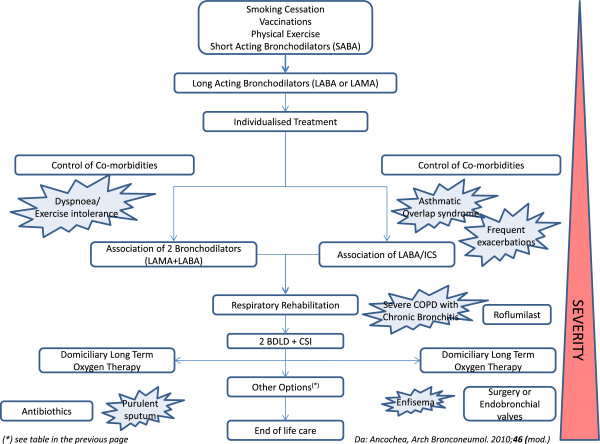
**A proposal for COPD treatment according to different stages of severity and different phenotypes.** From [113] mod.

In the advanced stages (very severe COPD), in addition to continuous oxygen therapy at home (alone in the case of isolated hypoxia, associated to noninvasive ventilation in the case of coexisting hypercapnia) antibiotics will be associated in the case of prevalent symptoms of chronic bronchitis with hypersecretions while in the case of a prevalent emphysematous component (localized, not generalized) the endoscopic application of valves will be evaluated. Figure 8[113] helps to position the single treatment components in relation to the patient’s level of severity (not, as said before, severity of bronchial obstruction only).

Finally, one cannot fail to mention the role of nutrition in patients affected by COPD, who encounter problems of malnutrition in two cases out of three. In these situations nutritional support can increase weight (as seen in the paragraph on staging of COPD, underweight is a negative prognostic factor in COPD), muscle mass, and fat mass as well as improve muscle strength [114].

#### How to organize treatment: physical exercise prescription and pulmonary rehabilitation

The reduction of physical exercise due to the dyspnea on exercise that is provoked by COPD, correlates directly with a significant decline in quality of life. This reduction often goes unnoticed inasmuch as the decline progresses slowly and in its turn it causes further exercise deconditioning, with dyspnea that appears at less and less demanding levels of physical exercise.

To combat both the sedentary lifestyle and its consequences it is necessary to recondition the organism to physical exercise through exercise training; the earlier the resumption of a reasonable level of physical activity the better the results will be. If in the initial phases of disease the patient can be left to self-manage the goals and means with a group treatment, e.g. through the walking groups organized by the Departments of Prevention of the community health service [115], in the moderate to severe phases it will be necessary to insert the patient in a structured program of Pulmonary Rehabilitation (PR) that will include not only exercise training but also nutritional assistance, psychological assistance, and education about the disease. But the first steps that have to be taken before starting exercise training are, as said before, smoking cessation (when the patient is an active smoker) and the optimization of bronchodilator therapy (see above). The contents of the PR program are summarized in Table 15 taken from the joint society document [35].

**Table 15:** Pulmonary rehabilitation

**Activities**	
**Essential activities**	**Optional activities**
Organize pharmacological treatments such as are required at the current point in time	Respiratory muscle training
Upper and/or lower limb muscle training	Chest physiotherapy
Health education	Nutritional support
Education about therapy	
Psychological and psycho-social support	

From [35].

Pulmonary rehabilitation is a multidisciplinary therapeutic intervention guided by the Pulmonologist and it can be administered both in an inpatient regime and as an outpatient service, or again in the patient’s home depending on the conditions of the patient and the facilities available. It needs furthermore to be practiced with regularity, given that its benefits tend to fade in the course of a few months to disappear completely after one year [116,117]. Besides being beneficial for the quality of life of the patient (it improves motivation and mood tone, rendering easier patient self-management = empowerment) PR, it seems, can also reduce exacerbations and hospitalizations [36,118]. Today in Italy, in the respiratory rehabilitation setting there exist almost only highly specialized centers, there being few community services, and it is necessary to increase the possibility of home-based rehabilitation. Nevertheless quite recently there has been a change in the organization of this sector of healthcare, and sports medicine has been given responsibility (for its greater competence in terms of physical exercise) in providing motor support for the primary and tertiary prevention of chronic degenerative diseases. And there are experiences of outpatient management of PR through this channel that have produced positive results [119].

#### How to organize treatment: use of oxygen therapy and ventilation therapy

Besides smoking cessation only long term oxygen therapy (LTOT) prescribed to people with COPD at a severe or very severe stage with partial arterial oxygen pressure lower than 55 or 60 mm/Hg (see below) has shown to be able to improve the survival of people affected by COPD, providing it is practiced daily for at least 15/18 hours. Besides survival there is a parallel improvement also in the quality of life and cognitive performance.

The need for oxygen therapy must obligatorily be established not only with pulse oximetry but also with blood gases analysis which provides information also on the carbon dioxide (and thus on the indication for eventual non-invasive ventilation therapy) and on the acid-base equilibrium. Noninvasive ventilation (which patients generally practice at night) should be assessed when the partial pressure of arterial carbon dioxide is chronically above 55 mm/Hg and/or rises dangerously if the hypoxia is corrected or again if there is present a disequilibrium of the acid-base condition of respiratory origin (pH > 7.35).

This treatment can be reserved, after specialist evaluation, for those who, besides hypercapnia, present frequent exacerbations and have required repeated hospitalizations [35]. As the prescription of non-invasive ventilation pertains strictly to specialists, we will not explore this issue in further depth here, just as we do not discuss here lung transplantation or volume reduction surgery and the endoscopic application of endobronchial valves, these latter being methods used, respectively, for the exeresis and desufflation of emphysematous areas. The criteria for initiating LTOT are reported in Table 16, taken from [35].

**Table 16:** Criteria for LTOT

●	PaO_2_< 7.3 KPa (55 mmHg, SaO_2_< 88%) in stable phase and during optimal therapeutic regime
●	PaO_2_ between 7.3 and 7.8 kPa (55-59 mmHg, SaO_2_< 89%) in the presence of pulmonary hypertension, pulmonary heart, declivity edema, erythrocytosis (hematocrit > 55%), cognitive deficit
●	Patients with manifest hypoxemia during exercise or at night

From [35].

**Figure 9:**
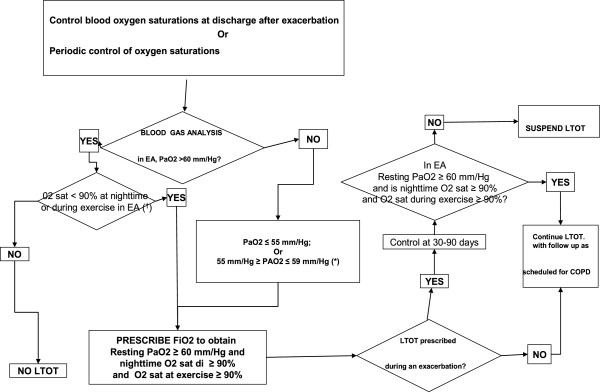
**Initial prescription and revised prescription of LTOT.** EA, Environmental air. (†) if the patient desaturates during exercise, in some studies, an improvement of exercise tolerance has been shown; without any effect on survival. From [36]. (*) in the presence of pulmonary hypertension, pulmonary heart, pitting edema, erythrocytosis (hematocrit < 55%), cognitive deficit. From [35].

The procedure for continuing or suspending LTOT is presented in Figure 9. The goal is to maintain O_2_ saturations always equal to or above 90%.

Oxygen therapy is available in various forms, from oxygen gas cylinders, to refrigerated liquid oxygen containers, to concentrators. The first finds practically no indication in pulmonary rehabilitation: its use is almost completely limited to emergency conditions or as a temporary supplementation of other sources when these are not available. Liquid O_2_ is versatile and allows mobility. Concentrators are indicated for patients confined to bed although, recently, portable concentrator devices have appeared. LTOT can be administered with nasal tube (more practical and less disturbing) or facial mask (mostly for significant flows or in special situations) or, in the case of tracheostomy, with an artificial nose [35].

A review of the LTOT prescription (when this occurs in the course of exacerbation and not due to the underlying disease progression) should always be programmed within a reasonably short time of the prescription itself in that the patient’s conditions can improve, making it superfluous, or can even worsen, making it useless for the prior-established goal. Both patient and family caregivers should be informed from the outset about the need for an eventual adjustment.

If there exist conditions for continuing LTOT, blood gas analyses should be included in the treatment plan, similarly as is done for smoking: a control is recommended at least once a year or in the event of any change in the patient’s clinical status (see section How to organize treatment: defining the disease severity). The frequency of controls varies from region to region according to protocols and norms issued by the regional health authorities, but in general they vary from 3 to 6 months [35].

## Notes on COPD care as the disease evolves

**Figure 10:**
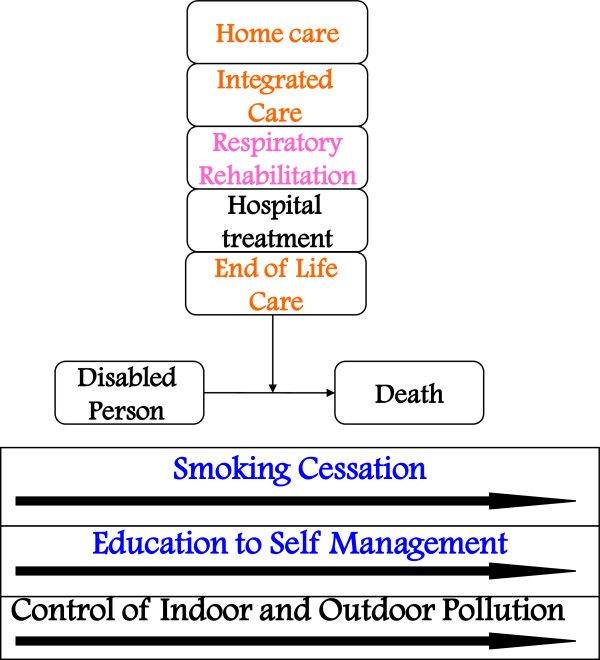
Ways to block the disease progression at the end of life.

In time, as the disease progresses the relative respiratory failure leads to invalidity and, subsequently, to death. Care modalities thus become necessary that - added to those outlined in the preceding paragraphs - assure the maximum possible well-being of the person through respiratory home care and, in advanced cases, end-of-life care (Figure 10).

On the other hand, chronic respiratory and cardiac diseases are among the most prevalent causes of invalidity, while dyspnoea is one of the most frequent (and invalidating) symptoms: hence home care constitutes a necessary support for the patients and their caregivers. The resulting burden for the National health service is not negligible, both in terms of the costs and the workload involved: Italian patients in LTOT still in 2009 were prudentially estimated to be about 100 per 100,000 inhabitants and to generate a cost for the NHS of more than 250 million annually [120].

### Homecare

In general the term ‘home care’ refers to a series of health treatments provided at the patient’s home rather than within a healthcare facility. There exist different (increasing) levels of home care, ranging from a simple control of the person’s general conditions and consumer goods supply (diapers, catheters, etc.) to specialized home care for ventilator-dependent persons. The latter care, which comes under the Pulmonary department of the hospital, consists in clinical controls and specialized functional monitoring and it constitutes the true respiratory home care.

In the last few years the concept of respiratory homecare has evolved to include not only the assistance described above, destined for patients already diagnosed and staged as to their severity and clinically stable, but also situations of instability due to acute events (e.g. exacerbations of COPD) not so severe as to require hospital admission (home care in this case supported by tele-assistance) [121]. Also the “protected” discharge from hospital of post-acute situations constitutes part of home care (discharge to home). This modality is also promoted by the Italian Ministry of Health in a system of integrated care. Discharge should always be agreed to prior with the GP, in the context of a formal, informed and agreed decision [122].

On this latter point, a further category of patients has recently been identified, namely “chronically critical” patients - these are people who have managed to survive what were, in fact, critical situations (e.g. a long stay in ICU) leaving them nevertheless subsequently with enduring states of organ failure and who, after a stay in the ICU or semi-intensive care followed by hospitalization in progressively less “intensive” wards, have arrived home with a need for mechanical ventilation and a very advanced level of invalidity. These are people who have before them months and sometimes years of stationary conditions. This new category of patients, rapidly on the rise numerically speaking, presents very slow progress (or regression) in their clinical conditions and a need for assistance by highly qualified personnel, and it represents a home care target that now is as inevitable as it is peculiar [123]. Here we will limit ourselves to the first type of respiratory home care described, i.e. that relative to known and stable patients, underlining however that, in the not too distant future, also the other activities described above will have to be included under the umbrella of ‘homecare’.

The scope of specialized respiratory home care is to monitor the situation through planned visits to the home, generally by a professional nurse with specialist training, or visits requested (generally by telephone) by the patient himself or by the GP. A check is made of symptoms, clinical status, and pulse oximetry. If considered necessary the nurse will perform a blood gases analysis. Symptoms and signs of clinical instability can be investigated by means of an outpatient visit and medical check. Exacerbations can be diagnosed early and treated at home or in the hospital depending on the clinical conditions and the family caregiver help available.

In Italy chronic respiratory patients are generally enrolled in the home care program when they become oxygen- or ventilator-dependent. In other countries, the enrolment includes also patients who have encountered in the preceding year frequent (> 2-3/year) exacerbations, independently of the presence of severe blood gas alterations [124]. But naturally this depends on the resources available.

Those to whom respiratory homecare is destined should also receive assistance to quit smoking (if current smokers) and education on the management of the advanced stage of their disease, including education about oxygen therapy. It is claimed that this mode of care, as well as improving the quality of life of patients in an advanced stage of COPD, also reduces their need for hospitalizations [124]. We referred to the role of the professional nurse in home care, but other figures too, if available, should be included in the home management team of these patients, as shown in Table 17, taken from the joint document and from the ministerial document of GARD-Italy on continuing care in COPD [35,122].

**Table 17:** Healthcare professionals involved in the home management of patients with respiratory failure

●	Referral doctor for integrated home care
●	“Specialized” professional nurse
●	Pulmonary rehabilitation therapist
●	Psychologist
●	Dietician/nutritionist

From [35,122].

These health professionals obviously should always be flanked by the patient’s GP, who constitutes the second level of reference and to whom the service of respiratory home care reports regarding the patient.

Home care today in Italy appears still far from completely established, it shows marked and significant variations between the different regions and, where it has been studied (perhaps as part of larger studies), poorly funded [80]. Instead, it ought to be implemented rapidly in order to respond to the needs arising not only from the increased numbers of those requiring care, but also from the progressive reduction of hospitals and the relative number of beds available.

### End-of-life care (palliative care)

Palliative care (which in the final phases becomes end-of-life care) consists in every intervention offered to ensure the patient the best possible quality of life, and a dignified death when this appears close or when the person affected by chronic respiratory disease does not respond any more to specific treatments. It does not offer to accelerate or delay death artificially. End-of-life care seeks to treat also the psycho-social aspects of the disease, including assistance to family members [125].

Essentially, palliative care consists in the remission of the symptoms afflicting the patient: dyspnea, abundant and/or difficult-to-clear sputum, pain, depression, anxiety, insomnia, various levels of immobility, and constipation. These symptoms are present also in terminal cancer patients and are treated in the same mode [125].

Controlling symptoms is not sufficient to guarantee the patient the best possible quality of life, if by quality of life one intends the difference between patients’ expectations and their real condition of health (i.e. Calman’s gap). While this latter is influenced by the capacity/possibility on the part of the palliative care team to contain symptoms and maximize the residual resources, the patient’s desires and expectations depend on their awareness of the incurability of their disease; thus communication of the truth or, better, accompanying the patient to understanding it represents one of the main tasks of the palliative doctor.

From an organizational point of view, the best response to the patient’s needs in an advanced phase of disease is provided through the network, which includes the dedicated, multidisciplinary palliative care team, (consisting of a pain care specialist, nurse, psychologist, social assistant, spiritual assistant, and others) that interacts with other physicians based in the hospital (pulmonologist) and local community, e.g. the GP and the continuing care physician [126].

Thus, for persons who have reached the final stage of disease, the group of professionals that is responsible for the care must include a pain care specialist. At this point, the role of the pulmonologist passes into the background, shifting from coordinator of activities (the role carried out in the overall activities of home care) to that of collaborator with the specialist responsible for pain care, who must not only possess skills in communication and human relations, but also knowledge about the treatment options in the advanced phases of the pathological processes, solid ethical notions and organizational capacities [127].

If, as said, the scope of pain care is to prevent and alleviate suffering, control symptoms and provide support to patients and their families in order to maintain and improve their quality of life, then - although pain care was originally conceived and implemented as an aid in approaching death - it should in fact be applied at all advanced stages of disease, both terminal and not, and should be available for patients at all stages of disease, targeted to their individual needs and requirements, as well as to the needs of their families, friends, and those who provide care as part of their job.

Clinicians who take care of patients with chronic or advanced respiratory disease or with critical diseases need to possess or acquire a series of basic skills in pain care. They should consult pain care specialists so as to be able to manage their patients in an appropriate manner under the supervision of other expert physicians. Also attention to grieving both before and after the patient’s death is recognized as an essential component of palliative care.

In this patient population, the difficulty in planning pain care resides in the unpredictability of the course of disease in the final phases, in that rarely are events heralded by a clear, unequivocal moment of transition and, on the other hand, what was up till this moment a very slow course can suddenly be precipitated by potentially fatal exacerbations [128].

These characteristics should orient the national health system toward greater attention to what the affected person effectively desires in terms of assistance and treatment. The uncertainty of the prognosis as regards survival means that, with respect to cancer patients, those affected by chronic respiratory disease will undergo far more frequently invasive interventions (from mechanical ventilation to cardiopulmonary resuscitation, to nutrition through naso gastric tube). Further, both persons in advanced cancer conditions and those with very severe chronic respiratory disease would appear to prefer the quality of their remaining life rather than simply a prolongation of their survival [129]. For this reason is has been suggested to anticipate the entry of COPD patients into palliative care, so they can be informed from the outset about the predicted course of the disease and state their preferences regarding assistance [127,130,131]. It hardly needs to be underlined that also in this setting the GP as always must be an indispensable part of the team [132].

## Conclusions

Primary and secondary prevention of COPD is recommended by the WHO through the model proposed by GARD. Also Italian ministerial documents and national and international guidelines recommend such a line of action. The end-point of the relative interventions is to improve community health and the care provided to patients with COPD in a way that is sustainable for the financial resources of the National Health Service.

On the basis of the WHO/GARD model, while Primary prevention is based on inter-sectorial interventions not centered on the healthcare system that aim to improve the living environment, eliminating or reducing the presence of gas and particles harmful to the air we breathe, Secondary prevention (i.e. early diagnosis) - while including actions and provisions not strictly pertaining to the health sector such as raising public awareness (advocacy) about pulmonary diseases and their most frequent symptoms - is composed instead of a series of actions that pertain specifically to healthcare.

Early diagnosis (an intervention which is today a priority in that it immediately reduces the social and individual consequences of COPD, while the effect of Primary prevention measures will not be seen until future years) could require greater investments in terms of organization, human and economic resources on the part of the national health service. Training of the healthcare personnel involved by scientific societies and the collection of data by these latter, that can demonstrate the efficiency and efficacy of the interventions adopted, would enable a correct planning of the changes that need to be made to respond to the effective needs and would facilitate their implementation.

However early diagnosis makes sense only if the system is able to follow up with an appropriate, standardized and sustainable treatment, something that requires not only the interdisciplinary collaboration of healthcare personnel but also the collaboration of patients (and their associations) who must know how and be able to self-manage their disease.

The challenge awaiting us in the future can be overcome only if all the components mentioned above can work together in a coordinated and synergic mode. Waiting for this coordination to come into full effect, we must not forget that the current priority in Italy is to collect and elaborate national data related not only to COPD epidemiology but also to the characteristics of its care, beginning with data on continuing care and on oxygen therapy.

## Endnotes

^a^For the meaning of these terms, see next paragraph “How to define bronchial obstruction”.

^b^It is beyond the scope of this document to discuss the greater sensitivity of the FEV_1_/VC ratio with respect to FEV_1_/FVC, or the merits for early diagnosis of examination of the curve pattern, or flow study at low lung volumes, or the measurement of residual volume as indices of obstruction.

^c^*By Treatment Education is intended the transmission of knowledge, training in performing skills and to promote changes in behavior, a transmission addressed to people affected by chronic disease, with the scope of improving the efficacy of treatment by means of their active and responsible participation in the program of care and assistance*[35]. In COPD the most important, though not the only, provision for treatment education concerns training in the correct use of inhaler devices (see section How to organize treatment: educating patients about their disease).

*Elaborated by the AIMAR Task-Force for the application of the WHO/GARD model on early diagnosis of COPD.

## Competing interests

The authors declare that they have no competing interests.

## References

[B1] http://www.un.org/ga/search/view_doc.asp?symbol=A/66/83&Lang=E

[B2] http://whqlibdoc.who.int/publications/2009/9789241597418_eng.pdf

[B3] http://www.who.int/gard/publications/GARD%20Book%202007.pdf

[B4] Improve healthcare by reducing unnecessary emergency admissionsn° 2006/0104, 20 Marzo 2006

[B5] Regione Veneto:Allegato “A” a DGR n^ 68/18, giugno 2013

[B6] Schiarire l'aria: uno studio nazionale sulla bronco pneumopatia cronica ostruttiva Multidiscip Resp Med 2007 2 1 19 22

[B7] Istituto Superiore di Sanità La mortalità per causa in Italia: 1980-2003 e 2006-2008 http://www.iss.it/site/mortalita/Scripts/SelCause.asp

[B8] De Marco R Accordini S Marcon A Cerveri I Antó JM Gislason T Heinrich J Janson C Jarvis D Kuenzli N Leynaert B Sunyer J Svanes C Wjst M Burney P European Community Respiratory Health Survey (ECRHS) Risk factors for chronic obstructive pulmonary disease in a European cohort of young adults Am J Respir Crit Care Med 2011 183 891 897 10.1164/rccm.201007-1125OC 20935112

[B9] Agenzia Nazionale per i Servizi Sanitari Regionali (AGENAS) Broncopneumopatia Cronica Ostruttiva - Linee guida nazionali di riferimento per la prevenzione e la terapia Roma 2011

[B10] Gini R Francesconi P Mazzaglia G Cricelli I Pasqua A Gallina P Brugaletta S Donato D Donatini A Marini A Zocchetti C Cricelli C Damiani G Bellentani M Sturkenboom MC Schuemie MJ Chronic disease prevalence from Italian administrative databases in the VALORE project: a validation through comparison of populationestimates with general practice databases and National survey BMC Public Health 2013 13 15 10.1186/1471-2458-13-15 23297821 PMC3551838

[B11] Viegi G Pedreschi M Pistelli F Di Pede F Baldacci S Carrozzi L Giuntini C Prevalence of airways obstruction in a general population. European Respiratory Society vs American Thoracic Society definition Chest 2000 117 5Suppl2 339S 345S 10843974 10.1378/chest.117.5_suppl_2.339s

[B12] Soverina P e gruppo SIMG-Pneumo La problematica previdenziale della broncopneumopatia cronica ostruttiva http://www.simg.it/documenti/rivista/2012/01_2012/8.pdf

[B13] Lindberg A Jonsson AC Rönmark E Lundgren R Larsson LG Lundbäck B Prevalence of chronic obstructive pulmonary disease according to BTS, ERS, GOLD and ATS criteria in relation to doctor's diagnosis, symptoms, age, gender, and smoking habits Respiration 2005 72 471 479 10.1159/000087670 16210885

[B14] Lindberg A Bjerg-Bäcklund A Rönmark E Larsson LG Lundbäck B Prevalence and underdiagnosis of COPD by disease severity and the attributable fraction of smoking. Report from the Obstructive Lung disease in Northern Sweden Studies Respir Med 2006 100 264 272 10.1016/j.rmed.2005.04.029 15975774

[B15] Dekhuijzen PN The Confronting COPD International Survey: patients hardly know they have COPD Eur Respir J 2002 20 793 794 10.1183/09031936.02.00404402 12412664

[B16] Beniwal S Sharma BB Singh V What we can say: disease illiteracy J Assoc Physicians India 2011 59 360 364 21751589

[B17] Murray CJ Lopez AD Regional patterns of disability-free life expectancy and disability-adjusted life expectancy: global Burden of disease Study Lancet 1997 349 1347 1352 10.1016/S0140-6736(96)07494-6 9149696

[B18] Shahab L Jarvis MJ Britton J West R Prevalence, diagnosis and relation to tobacco dependence of chronic obstructive pulmonary disease in a nationally representative population sample Thorax 2006 61 1043 1047 10.1136/thx.2006.064410 17040932 PMC2117062

[B19] Nardini S De Benedetto F Sanguinetti CM Donner CF per AIMAR (Associazione Scientifica Interdisciplinare per lo studio delle malattie Respiratorie) Il Progetto “SOS Respiro” di AIMAR. I risultati del primo progetto Italiano globale su COPD ed asma Multidiscip Resp Med 2007 2 4 16 23

[B20] Danielsson P Ólafsdóttir IS Benediktsdóttir B Gíslason T Janson C The prevalence of chronic obstructive pulmonary of disease in Uppsala, Sweden--the Burden of Obstructive Lung disease (BOLD) study: cross-sectional population-based study Clin Respir J 2012 6 2 120 127 10.1111/j.1752-699X.2011.00257.x 21651748

[B21] Rycroft CE Heyes A Lance L Becker K Epidemiology of chronic obstructive pulmonary disease: a literature review Int J Chron Obstruct Pulmon Dis 2012 7 457 494 22927753 10.2147/COPD.S32330 PMC3422122

[B22] Halbert RJ Isonaka S George D Iqbal A Interpreting COPD prevalence estimates: what is the true burden of disease? Chest 2003 123 5 1684 1692 10.1378/chest.123.5.1684 12740290

[B23] Halbert RJ Natoli JL Gano A Badamgarav E Buist AS Mannino DM Global burden of COPD: systematic review and meta-analysis Eur Respir J 2006 28 3 523 532 10.1183/09031936.06.00124605 16611654

[B24] Lindberg A Eriksson B Larsson LG Rönmark E Sandström T Lundbäck B Seven-year cumulative incidence of COPD in an age-stratified general population sample Chest 2006 129 4 879 885 10.1378/chest.129.4.879 16608933

[B25] http://www.patientsCOPD.it/pages/giornataMonofaleCOPD/download/Bettoncelli.pdf

[B26] Lusuardi M Orlandini D Han MK Martinez FJ Underutilization of spirometry for the diagnosis of COPD Chest 2008 133 313 314 10.1378/chest.07-2172 18187763

[B27] Lusuardi M Blasi F Terzano C Cricelli C Crispino N Comarella L De Benedetto F Sanguinetti CM Allegra L Donner CF Standards of care and clinical predictors in patients hospitalised for a COPD exacerbation - The Italian SOS Study (Stratification Observational Study) Monaldi Arch Chest Dis 2009 71 4,153-160 10.4081/monaldi.2009.347 20440919

[B28] Piperno D Huchon G Pribil C Boucot I Similowski T The burden of COPD in France: results from the Confronting COPD survey Respir Med 2003 97 Suppl C S33 S42 12647941 10.1016/s0954-6111(03)80023-9

[B29] British Thoracic Society The Burden of Lung Diseases- 2007 http://www.brit-thoracic.org.uk/Portals/0/Library/BTS%20Publications/burden_of_lung_ofsease.pdf

[B30] http://www.cesfar.it/farmacoeconomia/3-analisi-economica-dei-costi-correlati-alla-gestione dei pazienti in ossigeno terapia domiciliare a lungo termine (OTLT) con o senza monitoraggio telemetrico domiciliare.html

[B31] http://www.rssp.salute.gov.it/rssp2011/paginaParagrafoRssp2011.jsp?setion?=?analisi&capitolo?=?quadro&id?=?3149)

[B32] 10 http://www.gov.uk/government/uploads/system/uploads/attachment_data/file/213840/dh_113279.pdf

[B33] http://www.salute.gov.it/imgs/C_17_pubblicazioni_1206_allegato.pdf

[B34] http://www.salute.gov.it/imgs/C_17_pubblicazioni_1893_allegato.pdf

[B35] http://www.aimarnet.it/wordpress/wp-content/uploads/2013/02/ManagementCOPD.pdf

[B36] http://www.agenas.it/images/agenas/pnlg/BPCO.pdf

[B37] http://www.iss.it/binary/fumo/cont/carte_del_risk_COPD_e_TaP.pdf

[B38] http://drfosterintelligence.co.uk/wp-content/uploads/2013/02/Hospital_Guide_2012.pdf).

[B39] http://www.who.int/quantifying_ehimpacts/publications/preventingofsease.pdf

[B40] http://www.who.int/fctc/en/index.html

[B41] http://www.who.int/fctc/reporting/progress_report_final.pdf

[B42] http://www.ccm-network.it/documenti_Ccm/convegni/SANIT/materiali2008/25.6/1-Promotion_salute_Galeone.pdf

[B43] http://www.epicentro.iss.it/temi/materno/rapporto_europa.asp

[B44] http://www.salute.gov.it/imgs/C_17_pubblicazioni_1454_allegato.pdf

[B45] Miravitlles M de la Roza C Morera J Montemayor T Gobartt E Martín A Alvarez-Sala JL Chronic respiratory symptoms, spirometry and knowledge of COPD among general population Respir Med 2006 100 11 1973 1980 10.1016/j.rmed.2006.02.024 16626950

[B46] Rabe KF Hurd S Anzueto A Barnes PJ Buist SA Calverley P Fukuchi Y Jenkins C Rodriguez-Roisin R van Weel C Zielinski J Global Initiative for Chronic Obstructive Lung Ofsease Global strategy for the diagnosis, management, and prevention of chronic obstructive pulmonary disease: GOLD executive summary Am J Respir Crit Care Med 2007 176 6 532 555 10.1164/rccm.200703-456SO 17507545

[B47] Van Schayck CP Loozen JMC Wagena E Akkermans RP Wesseling GJ Detecting patients at a high risk of developing chronic obstructive pulmonary disease in general practice: cross sectional case finding study BMJ 2002 324 1370 10.1136/bmj.324.7350.1370 12052807 PMC115215

[B48] Tønnesen P Smoking cessation and COPD Eur Respir Rev 2013 22 127 37 43 10.1183/09059180.00007212 23457163 PMC9487432

[B49] Alderson SL Foy R Glidewell L McLintock K House A How patients understand depression associated with chronic physical disease – a systematic review BMC Fam Pract 2012 13 41 10.1186/1471-2296-13-41 22640234 PMC3439302

[B50] Petty TL Definitions, causes, course, and prognosis of chronic obstructive pulmonary disease Respir Care Clin N Am 1998 4 345 358 9770256

[B51] Rudolf M The reality of drug use in COPD: the European perspective Chest 2000 117 2 suppl 29S 32S 10673471 10.1378/chest.117.2_suppl.29s

[B52] Calverley PM Nordyke RJ Halbert RJ Isonaka S Nonikov D Development of a population-based screening questionnaire for COPD COPD 2005 2 2 225 232 10.1081/COPD-57594 17136949

[B53] van Schayck CP Halbert RJ Nordyke RJ Isonaka S Maroni J Nonikov D Comparison of existing symptom-based questionnaires for identifying COPD in the general practice setting Respirology 2005 10 3 323 333 10.1111/j.1440-1843.2005.00720.x 15955145

[B54] Freeman D Nordyke RJ Isonaka S Nonikov DV Maroni JM Price D Halbert RJ Questions for COPD diagnostic screening in a primary care setting Respir Med 2005 99 10 1311 1318 10.1016/j.rmed.2005.02.037 16140231

[B55] Mapel DW Frost FJ Hurley JS Petersen H Roberts M Marton JP Shah H An algorithm for the identification of undiagnosed COPD cases using administrative claims data J Manag Care Pharm 2006 12 6 457 465 16925453 10.18553/jmcp.2006.12.6.458 PMC10438324

[B56] Price DB Tinkelman DG Nordyke RJ Isonaka S Halbert RJ COPD Questionnaire Study Group Scoring system and clinical application of COPD diagnostic questionnaires Chest 2006 129 6 1531 1539 10.1378/chest.129.6.1531 16778271

[B57] Nelson SB LaVange LM Nie Y Walsh JW Enright PL Martinez FJ Mannino DM Thomashow BM Questionnaires and pocket spirometers provide an alternative approach for COPD screening in the general population Chest 2012 142 2 358 366 22194590 10.1378/chest.11-1474

[B58] Hegewald MJ Lefor MJ Jensen RL Crapo RO Kritchevsky SB Haggerty CL Bauer DC Satterfield S Harris T Peak expiratory flow is not a quality indicator for spirometry: peak expiratory flow variability and FEV1 are poorly correlated in an elderly population Chest 2007 131 1494 1499 10.1378/chest.06-2707 17400677

[B59] http://www.drive4copd.org/AreYouAtRisk/TaketheScreener.aspx

[B60] Martinez FJ Raczek AE Seifer FD Conoscenti CS Curtice TG D'Eletto T Cote C Hawkins C Phillips AL COPD-PS Clinician Working Group Development and initial validation of a self-scored COPD Population Screener Questionnaire (COPD-PS) COPD 2008 5 2 85 95 http://www.ncbi.nlm.nih.gov/pmc/articles/PMC2430173/pdf/lcpd5-85.pdf 10.1080/15412550801940721 18415807 PMC2430173

[B61] Dirven JA Tange HJ Muris JW van Haaren KM Vink G van Schayck OC Early detection of COPD in general practice: patient or practice managed? A randomised controlled trial of two strategies in different socioeconomic environments Prim Care Respir J 2013 22 3 331 337 10.4104/pcrj.2013.00070 23966214 PMC6442827

[B62] Stratelis G Jakobsson P Molstad S Zetterstrom O Early detection of COPD in primary care: screening by invitation of smokers aged 40 to 55 years Br J Gen Pract 2004 54 500 201 206 15006126 PMC1314831

[B63] Zuccaro P Pichini S Mortali C Pacifici R Viegi G Baldacci S Angino A Martini F Borbotti M Scognamiglio A Simoni M Silvi P DiPede F Carrozzi L Porta D Simonato L Crispo A Merletti F Forastiere F Fumo e patologie respiratorie: le carte del rischio per COPD e tumore al polmone Multidiscip Resp Med 2007 2 14 21

[B64] http://www.iss.it/binary/fumo4/cont/carte_del_rischio_BPCO_e_TaP.pdf

[B65] http://www.salute.gov.it/imgs/C_17_pubblicazioni_1891_allegato.pdf

[B66] Ferro A Cinquetti S Moro A Siddu A Trimarchi A Penon MG Pavan PP Camillotto R Rossetto L Volpe V Zevrain S Brusaferro S Prevenire le patologie cardiovascolari attraverso un modello di valutazione proattiva del rischio (screening) applicabile ad ampie fasce di popolazione. Risultati della prima fase del progetto Epidemiologia e Prevenzione 2013 in press 24736960

[B67] Cawley MJ Moon J Reinhold J Willey VJ Warning Ii WJ Spirometry: tool for pharmacy practitioners to expand direct patient care services J Am Pharm Assoc (2003) 2013 53 3 307 315 10.1331/JAPhA.2013.12134 23699680

[B68] Miller MR Hankinson J Brusasco V Burgos F Casaburi R Coates A Crapo R Enright P van der Grinten CP Gustafsson P Jensen R Johnson DC MacIntyre N McKay R Navajas D Pedersen OF Pellegrino R Viegi G Wanger J ATS/ERS Task Force Standardisation of spirometry Eur Respir J 2005 26 2 319 338 10.1183/09031936.05.00034805 16055882

[B69] Vandevoorde J Verbanck S Schuermans D Kartounian J Vincken W FEV1/FEV6 and FEV6 as an alternative for FEV1/FVC and FVC in the spirometric detection of airway obstruction and restriction Chest 2005 127 1560 1564 10.1378/chest.127.5.1560 15888828

[B70] Korhonen H Remes ST Kannisto S Korppi M Hand-held turbine spirometer: agreement with the conventional spirometer at baseline and after exercise Pediatr Allergy Immunol 2005 16 3 254 257 10.1111/j.1399-3038.2005.00252.x 15853956

[B71] Schermer TR Verweij EH Cretier R Pellegrino A Crockett AJ Poels PJ Accuracy and precision of desktop spirometers in general practices Respiration 2012 83 4 344 352 10.1159/000334320 22236628

[B72] Ofrksen A Madsen F Pedersen OF Vedel AM Kok-Jensen A Long term performance of a hand held spirometer Thorax 1996 51 973 976 10.1136/thx.51.10.973 8977594 PMC472642

[B73] Malmberg LP Hedman J Sovijärvi AR Accuracy and repeatability of a pocket turbine spirometer: comparison with a rolling seal flow-volume spirometer Clin Physiol 1993 13 1 89 98 10.1111/j.1475-097X.1993.tb00320.x 8435980

[B74] Enright PL How to make sure your Spirometry Tests are of good quality Respir Care 2003 48 8 773 776 12890297

[B75] Dowson LJ Yeung A Allen MB General practice spirometry in North Staffordshire Monaldi Arch Chest Dis 1999 54 2 186 188 10394838

[B76] Bracci M Strafella E Croce N Staffolani S Carducci A Verani M Valentino M Santarelli L Risk of bacterial cross infection associated with inspiration through flow-based spirometers Am J Infect Control 2011 39 1 50 55 10.1016/j.ajic.2010.04.215 20817316

[B77] Lusuardi M De Benedetto F Paggiaro P Sanguinetti CM Brazzola G Ferri P Donner CF A randomized controlled trial on office spirometry in asthma and COPD in standard general practice: data from spirometry in Asthma and COPD: a comparative evaluation Italian study Chest 2006 129 4 844 852 10.1378/chest.129.4.844 16608929

[B78] Enright P The use and abuse of office spirometry Prim Care Respir J 2008 17 4 238 242 10.3132/pcrj.2008.00065 18958360 PMC6619909

[B79] Enright P Provide GPs with spirometry not spirometers Thorax 2008 63 5 387 388 10.1136/thx.2007.092916 18443153

[B80] Nardini S Cicchitto G De Benedetto F Donner CF Polverino M Sanguinetti CM Visconti A Audit sull’ appropriatezza nell’assistenza integrata della BPCO. Il progetto “ALT-BPCO” Multidiscip Resp Med 2012 7 5-6 I XII

[B81] Culver BH How Should the Lower Limit of the Normal Range Be Defined? Respir Care 2012 57 136 145 10.4187/respcare.01427 22222132

[B82] Pellegrino R Viegi G Brusasco V Crapo RO Burgos F Casaburi R Coates A van der Grinten CPM Gustafsson P Hankinson J Jensen R Johnson DC MacIntyre N McKay R Miller MR Navajas D Pedersen OF Wanger J Interpretative strategies for lung function tests Eur Respir J 2005 26 948 968 10.1183/09031936.05.00035205 16264058

[B83] http://www.goldcopd.it/traduzioni2011/GOLDREPORT2011Ita.pdf

[B84] Celli BR Halbert RJ Isonaka S Schau B Population impact of different definitions of airway obstruction Eur Respir J 2003 22 2 268 273 10.1183/09031936.03.00075102 12952259

[B85] Mohamed Hoesein FA Zanen P Lammers JW Lower limit of normal or FEV1/FVC < 0.70 in diagnosisng COPD: an evidence-based review Respir Med 2011 105 6 907 915 10.1016/j.rmed.2011.01.008 21295958

[B86] National Institute for Clinical Excellence (NICE) Clinical Guideline Centre Chronic Obstructive Pulmonary Disease: Management of Chronic Obstructive Pulmonary Disease in Adults in Primary and Secondary Care. Update Guideline 2011 72 74

[B87] Nishimura K Izumu T Tsukino M Oga T Dyspnea is a better predictor of 5-year survival than airway obstruction in patients with COPD Chest 2002 121 1434 1440 10.1378/chest.121.5.1434 12006425

[B88] Celli BR Cote CG Marin JM Casanova C Montes de Oca M Méndez RA Pinto Plata V Cabral HJ The body-mass index, airflow obstruction, dyspnea, and exercise capacity index in chronic obstructive pulmonary disease N Engl J Med 2004 350 1005 1012 10.1056/NEJMoa021322 14999112

[B89] Anzueto A Leimer I Kesten S Impact of frequency of COPD exacerbations on pulmonary function, health status and clinical outcomes Int J COPD 2009 4 245 251 10.2147/copd.s4862 PMC2719254 19657398

[B90] Sullivan SD Buist AS Weiss K Health outcome assessment and economic evaluation in COPD: challenges and opportunities Eur Respir J 2003 21 suppl.41 1s 3s 10.1183/09031936.03.00077603 12795325

[B91] Effing T Monninkhof EEM van der Valk PD van der Palen J van Herwaarden CL Partridge MR Walters EH Zielhuis GA Self-management education for patients with chronic obstructive pulmonary disease Cochrane Database Syst Rev 2007 4 CD002990 doi:10.1002/14651858.CD002990.pub2 17943778 10.1002/14651858.CD002990.pub2

[B92] Adams SG Smith PK Allan PF Anzueto A Pugh JA Cornell JE Systematic review of the chronic care model in chronic obstructive pulmonary disease prevention and management Arch Intern Med 2007 167 6 551 561 10.1001/archinte.167.6.551 17389286

[B93] Rice KL Dewan N Bloomfield HE Grill J Schult TM Nelson DB Kumari S Thomas M Geist LJ Beaner C Caldwell M Niewoehner DE Disease management program for chronic obstructive pulmonary disease: a randomized controlled trial Am J Respir Crit Care Med 2010 182 7 890 896 10.1164/rccm.200910-1579OC 20075385

[B94] Walters JAE Turnock AC Walters EH Wood-Baker R Action plans with limited patient education only for exacerbations of chronic obstructive pulmonary disease Cochrane Database Syst Rev 2010 5 CD005074 doi:10.1002/14651858.CD005074.pub3 20464737 10.1002/14651858.CD005074.pub3

[B95] McLean S Nurmatov U Liu JLY Pagliari C Car J Sheikh A Telehealthcare for chronic obstructive pulmonary disease Cochrane Database Syst Rev 2011 7 CD007718 doi:10.1002/14651858.CD007718.pub2 21735417 10.1002/14651858.CD007718.pub2 PMC8939044

[B96] Koff PB Jones RH Cashman JM Voelkel NF Vanofvier RW Proactive integrated care improves quality of life in patients with COPD Eur Respir J 2009 33 5 1031 1038 10.1183/09031936.00063108 19129289

[B97] Clark NM Dodge JA Partridge MR Martinez FJ Focusing on outcomes: making the most of COPD interventions Int J Chron Obstruct Pulmon Dis 2009 4 61 77 19436690 PMC2672794

[B98] Lareau SC Yawn BP Improving adherence with inhaler therapy in COPD Int J Chron Obstruct Pulmon Dis 2010 5 401 406 21191434 10.2147/COPD.S14715 PMC3008325

[B99] Yawn BP Colice GL Hodder R Practical aspects of inhaler use in the management of chronic obstructive pulmonary Disease in the primary care setting Int J Chron Obstruct Pulmon Dis 2012 7 495 502 22888221 10.2147/COPD.S32674 PMC3413176

[B100] Tønnesen P Carrozzi L Fagerstroem KO Gratziou C Jimenez-Ruiz C Nardini S Viegi G Lazzaro C Campell IA Dagli E West R Smoking cessation in patients with respiratory diseases: a high priority, integral component of therapy Eur Respir J 2007 29 390 417 17264326 10.1183/09031936.00060806

[B101] Górecka D Bednarek M Nowiński A Puścińska E Goljan-Geremek A Zieliński J Diagnosis of airflow limitation combined with smoking cessation advice increases stop-smoking rate Chest 2003 123 6 1916 1923 10.1378/chest.123.6.1916 12796168

[B102] Stratelis G Mölstad S Jakobsson P Zetterström O The impact of repeated spirometry and smoking cessation advice on smokers with mild COPD Scand J Prim Health Care 2006 24 3 133 139 10.1080/02813430600819751 16923621

[B103] Fiore M Jaen CR Baker TB Bailey WC Benowitz N Curry SJ Dorfman SF Froelicher ES Goldstein MG Healton CG Henderson PN Heyman RB Koh HK Kottke TE Lando HA Mecklenburg RE Mermelstein RJ Mullen PD Orleans CT Robinson L Stitzer ML Tommasello AC Villejo L Wewers ME Murray EW Bennett G Heishman S Husten C Morgan G Williams C Christiansen BA Piper ME Hasselblad V Fraser D Theobald W Connell M Leitzke C Treating tobacco use and dependence: 2008 update. U.S. Public Health Service Clinical Practice Guideline executive summary Respir Care 2008 53 1217 1222 18807274

[B104] Guida ai servizi territoriali per la cessazione dal fumo of tabacco (aggiornamento 2012). A cura dell’Osservatorio Fumo, Alcol e Droga 2013 http://www.iss.it/publ/index.php?lang?=?1&id?=?2707&tipo?=?7

[B105] Hughes JR Carpenter MJ The feasibility of smoking reduction: an update Addiction 2005 100 8 1074 1089 10.1111/j.1360-0443.2005.01174.x 16042638 PMC1419056

[B106] Lindson-Hawley N Aveyard P Hughes JR Reduction versus abrupt cessation in smokers who want to quit Cochrane Database Syst Rev 2012 11 CD008033 23152252 10.1002/14651858.CD008033.pub3

[B107] Moore D Aveyard P Connock M Wang D Fry-Smith A Barton P Effectiveness and safety of nicotine replacement therapy assisted reduction to stop smoking: systematic review and meta-analysis BMJ 2009 338 b1024 10.1136/bmj.b1024 19342408 PMC2664870

[B108] Lee PN The effect of reducing the number of cigarettes smoked on risk of lung cancer, COPD, cardio-vascular disease and FEV_1_ - a review Regul Toxicol Pharmacol 2013 67 372 381 10.1016/j.yrtph.2013.08.016 24013038

[B109] Nardini S Pacifici R E-cigarettes, smokers and health policies Monaldi Arch Chest Dis 2013 79 1 6 7 23741939 10.4081/monaldi.2013.102

[B110] Bateman E Vogelmeier C Chen H Banerji D Comparison of COPD exacerbations with once-daily QVA149 versus twice-daily Salmeterol/Fluticasone Combination: The ILLUMINATE Study Chest 2014 145 3 Suppl 409A doi:10.1378/chest.1824338

[B111] Wedzicha J Ficker J Fowlertaylor A D'Andrea P Arrasate C Chen H Banerji D Once-Daily QVA149 reduces exacerbations and improves health status in comparison with glycopyrronium and tiotropium in patients with severe-to-very severe COPD: The SPARK Study Chest 2014 145 3 Suppl 427A doi:10.1378/chest.182441424493538

[B112] Cosio BG Tsaprouni L Ito K Jazrawi E Adcock IM Barnes PJ Theophylline restores histone deacetylase activity and steroid responses in COPD macrophages J Exp Med 2004 200 689 695 10.1084/jem.20040416 15337792 PMC2212744

[B113] Ancochea J Gómez García T de Miguel DJ Hacia un tratamiento individualizado e integrado del paciente con EPOC Arch Bronconeumol 2010 46 Suppl 10 14 18 21316551 10.1016/S0300-2896(10)70051-X

[B114] Collins PF Stratton RJ Elia M Nutritional support in chronic obstructive pulmonary disease: a systematic review and meta-analysis Am J Clin Nutr 2012 95 1385 1395 10.3945/ajcn.111.023499 22513295

[B115] http://www.epicentro.iss.it/problemi/obesita/pdf/territorio/lombardia/milano/Manuale%20WL%20small.pdf

[B116] Nici L Donner C Wouters E Zuwallack R Ambrosino N Bourbeau J Carone M Celli B Engelen M Fahy B Garvey C Goldstein R Gosselink R Lareau S MacIntyre N Maltais F Morgan M O'Donnell D Prefault C Reardon J Rochester C Schols A Singh S Troosters T ATS/ERS Pulmonary Rehabilitation Writing Committee: American Thoracic Society/European Respiratory Society statement on pulmonary rehabilitation Am J Respir Crit Care Med 2006 173 12 1390 1413 10.1164/rccm.200508-1211ST 16760357

[B117] Ries AL Bauldoff GS Carlin BW Casaburi R Emery CF Mahler DA Make B Rochester CL Zuwallack R Herrerias C Pulmonary rehabilitation: Joint ACCP/AACVPR evidence-based clinical practice guidelines Chest 2007 131 suppl 5 4S 42S 17494825 10.1378/chest.06-2418

[B118] Puhan MA Gimeno-Santos E Scharplatz M Troosters T Walters EH Steurer J Pulmonary rehabilitation following exacerbations of chronic obstructive pulmonary disease Cochrane Database Syst Rev 2011 10 CD005305 21975749 10.1002/14651858.CD005305.pub3

[B119] Personal Observations

[B120] http://www.isde.it/iniziative/2009/Novembre-Salsomaggiore/4/Corrado%20presentation.pdf

[B121] McCurdy BR Hospital-at-home programs for patients with acute exacerbations of chronic obstructive pulmonary disease (COPD): an evidence-based analysis Ont Health Technol Assess Ser 2012 12 1 65 PMC3384361 23074420

[B122] GARD-Italy La continuità assistenziale: Broncopneumopatia Cronica Ostruttiva (COPD) 2011 Roma http://www.salute.gov.it/imgs/C_17_pubblicazioni_1893_allegato.pdf

[B123] Macintyre NR Chronic critical illness: the growing challenge to health care Respir Care 2012 57 6 1021 1027 10.4187/respcare.01768 22663975

[B124] American Thoracic Society Documents Statement on Home Care for Patients with Respiratory Disorders Am J Respir Crit Care Med 2005 171 1443 1464 15941843 10.1164/rccm.2504001

[B125] http://www.siaarti.it/documenti/pdf_doc/file_15.pd

[B126] Legge n°38 del 2010 “Disposizioni per garantire l’accesso alle cure palliative e alla terapia del dolore”- Intesa Stato-Regioni del 25 luglio 2012 “Requisiti minimi organizzativi per l’accreditamento delle reti di cure palliative e di terapia del dolore

[B127] Lanken PN Terry PB DeLisser HM Fahy BF Hansen-Flaschen J Heffner JE Levy M Mularski RA Osborne ML Prendergast TJ Rocker G Sibbald WJ† Wilfond B Yankaskas JR on behalf of the ATS End-of-Life Care Task Force An Official American Thoracic Society Clinical Policy Statement: Palliative Care for Patients with Respiratory Diseases and Critical Illnesses Am J Respir Crit Care Med 2008 177 912 927 10.1164/rccm.200605-587ST 18390964

[B128] Murray SA Kendall M Boyd K Sheikh A Illness trajectories and palliative care BMJ 2005 330 1007 1011 10.1136/bmj.330.7498.1007 15860828 PMC557152

[B129] Claessens MT Lynn J Zhong Z Desbiens NA Phillips RS Wu AW Harrell FE Jr Connors AF Jr Dying with lung cancer or chronic obstructive pulmonary Disease: insights from SUPPORT. Study to understand prognoses and preferences for outcomes and risks of treatments J Am Geriatr Soc 2000 48 S146 S153 10809468 10.1111/j.1532-5415.2000.tb03124.x

[B130] Carlucci A Guerrieri A Nava S Palliative care in COPD patients: is it only an end-of-life issue? Eur Respir Rev 2012 21 126 347 354 10.1183/09059180.00001512 23204123 PMC9487222

[B131] Goodridge DM Marciniuk DD Brooks D van Dam A Hutchinson S Bailey P Baxter S Dorasamy P Dumont S Hassan S Hernandez P Kerigan A Rocker G Wilson D Young J End-of-life care for persons with advanced chronic obstructive pulmonary disease: report of a national interdisciplinary consensus meeting Can Respir J 2009 16 5 e51 e53 19851529 10.1155/2009/987616 PMC2779173

[B132] Freeman D Price D ABC of chronic obstructive pulmonary disease. Primary care and palliative care BMJ 2006 333 188 190 10.1136/bmj.333.7560.188 16858049 PMC1513460

